# Hepatitis B surface antigen reduction is associated with hepatitis B core-specific CD8^+^ T cell quality

**DOI:** 10.3389/fimmu.2023.1257113

**Published:** 2023-10-18

**Authors:** Shokichi Takahama, Sachiyo Yoshio, Yuji Masuta, Hirotomo Murakami, Ryotaro Sakamori, Shun Kaneko, Takashi Honda, Miyako Murakawa, Masaya Sugiyama, Masayuki Kurosaki, Yasuhiro Asahina, Tetsuo Takehara, Victor Appay, Tatsuya Kanto, Takuya Yamamoto

**Affiliations:** ^1^ Laboratory of Precision Immunology, Center for Intractable Diseases and ImmunoGenomics, National Institutes of Biomedical Innovation, Health and Nutrition, Osaka, Japan; ^2^ Department of Liver Diseases, Research Center for Hepatitis and Immunology, National Center for Global Health and Medicine, Chiba, Japan; ^3^ Department of Gastroenterological Surgery, Graduate School of Medicine, Osaka University, Osaka, Japan; ^4^ Department of Gastroenterology and Hepatology, Osaka University Graduate School of Medicine, Osaka, Japan; ^5^ Department of Gastroenterology and Hepatology, Musashino Red Cross Hospital, Tokyo, Japan; ^6^ Department of Gastroenterology and Hepatology, Tokyo Medical and Dental University, Tokyo, Japan; ^7^ Department of Gastroenterology and Hepatology, Nagoya University Graduate School of Medicine, Nagoya, Japan; ^8^ Department of Viral Pathogenesis and Controls, National Center for Global Health and Medicine, Tokyo, Japan; ^9^ Department of Liver Disease Control, Tokyo Medical and Dental University, Tokyo, Japan; ^10^ Université de Bordeaux, CNRS, Institut national de la santé et de la recherche médicale (INSERM), ImmunoConcEpT, UMR 5164, Bordeaux, France; ^11^ Laboratory of Translational Cancer Immunology and Biology, Next-generation Precision Medicine Research Center, Osaka International Cancer Institute, Osaka, Japan; ^12^ The Research Institute for Microbial Diseases, Osaka University, Osaka, Japan; ^13^ Department of Virology and Immunology, Graduate School of Medicine, Osaka University, Osaka, Japan

**Keywords:** Chronic hepatitis B, HBsAg, HBV-specific CD8 + T cells, scRNA-seq, cytotoxicity

## Abstract

Despite treatment, hepatitis B surface antigen (HBsAg) persists in patients with chronic hepatitis B (CHB), suggesting the likely presence of the virus in the body. CD8^+^ T cell responses are essential for managing viral replication, but their effect on HBsAg levels remains unclear. We studied the traits of activated CD8^+^ T cells and HBV-specific CD8^+^ T cells in the blood of CHB patients undergoing nucleos(t)ide analog (NUC) therapy. For the transcriptome profiling of activated CD8^+^ T cells in peripheral blood mononuclear cells (PBMCs), CD69^+^ CD8^+^ T cells were sorted from six donors, and single-cell RNA sequencing (scRNA-seq) analysis was performed. To detect HBV-specific CD8^+^ T cells, we stimulated PBMCs from 26 donors with overlapping peptides covering the HBs, HBcore, and HBpol regions of genotype A/B/C viruses, cultured for 10 days, and analyzed via multicolor flow cytometry. scRNA-seq data revealed that CD8^+^ T cell clusters harboring the transcripts involved in the cytolytic functions were frequently observed in donors with high HBsAg levels. Polyfunctional analysis of HBV-specific CD8^+^ T cells utilized by IFN-γ/TNFα/CD107A/CD137 revealed that HBcore-specific cells exhibited greater polyfunctionality, suggesting that the quality of HBV-specific CD8^+^ T cells varies among antigens. Moreover, a subset of HBcore-specific CD8^+^ T cells with lower cytolytic potential was inversely correlated with HBsAg level. Our results revealed a stimulant-dependent qualitative difference in HBV-specific CD8^+^ T cells in patients with CHB undergoing NUC therapy. Hence, the induction of HBcore-specific CD8^+^ T cells with lower cytolytic potential could be a new target for reducing HBsAg levels.

## 1 Introduction

Chronic hepatitis B (CHB) virus (HBV) infection induces immunopathology, leading to chronic hepatitis, fibrosis, and eventually to cirrhosis and hepatocellular carcinoma. Millions of people are chronically infected and diagnosed with hepatitis B, resulting in approximately one million deaths yearly (1). Attempts to reduce the viral load via treatment with PEGylated interferons or nucleos(t)ide analogs (NUCs) have improved the quality of life of patients with CHB. It is possible to efficiently suppress HBV replication and reduce liver inflammation using NUCs. In addition, Rivino et al. show the possible contribution of HBV-specific T cells for the sustained viral control in a group of patients who discontinued antiviral drug treatment (2). However, generally, they neither eliminate all virus-infected cells nor achieve sustained virologic control after treatment discontinuation. Hepatitis B surface antigens (HBsAgs) are persistently detected in many NUC-treated patients, and HBsAg levels are correlated with the risk of carcinogenesis (3, 4). Therefore, lowering HBsAg levels in the body is necessary to achieve a functional cure for HBV infection.

Conversely, cellular immunity, including antigen-specific CD8^+^ T cells, is important for eliminating virus-infected cells (5, 6). In an experimental model of acute HBV infection in chimpanzees, CD8^+^ T cell infiltration into the liver precedes viral clearance (7). The induction of multifunctional T cells is also essential because multifunctional T cell immunity is key in viral suppression in acute hepatitis (8). In contrast to acute infection, HBV-specific CD8^+^ T cell responses during chronic persistent infection are low (9, 10), and antigen-specific CD8^+^ T cells are exhausted (11). However, activated CTL responses are maintained in patients recovering from CHB (12). HBV-specific T cell function is improved in individuals with HBsAg loss, despite the high expression of exhaustion-related molecules such as TOX and PD-1 (13). HBV-specific T cells are involved in eliminating covalently closed circuit DNA (cccDNA) from the body *in vivo* (14). Importantly, HBs-specific T cells are not detected in patients recovering from CHB, unlike HBcore- and HBpol-specific T cells (15), suggesting qualitative differences in HBV-specific T cells induced by each HBV antigen. The importance of HBV-specific CD8^+^ T cells upon discontinuing NUC therapy has also been reported, with PD-1-high expressing HBcore- and HBpol-specific T cells being more functional than PD-1-negative cells (2). Among HBeAg-negative patients with CHB, HBsAg loss was observed in 30% of patients who discontinued NUC treatment. In that case, the frequency of HBV-specific CD8^+^ T cells before treatment discontinuation has been suggested to contribute to viral control (16). In addition, there were more cases of disappearance in patients with HBsAg levels below 1000 U/mL before treatment discontinuation (16). Specimens from patients who discontinued NUC treatment responded to multiple HBV-derived antigens, particularly IFN-γ- and TNFα-producing cells, which vanished upon resumption of NUC treatment. Although the importance of HBV-specific CD8^+^ T cells for HBsAg control under NUC treatment has been suggested as above, reports on the characterization of qualitative differences in these antigen-specific CD8^+^ T cells are limited.

In this study, we first characterized activated CD8^+^ T cells in the peripheral blood mononuclear cells (PBMCs) of patients with CHB under NUC treatment by performing single-cell RNA sequencing (scRNA-seq) on CD69^+^-sorted CD8^+^ T cells. Gene expression profiles were determined to distinguish cell populations with different functional profiles, which were then examined in relation to HBsAg levels; lower HBsAg levels were associated with a higher frequency of the cell population that was considered to have weak cytotoxic activity. Furthermore, flow cytometry analysis revealed that the quality of HBcore-specific CD8^+^ T cells related to the production of IFN-γ and TNFα, but not CD107A and CD137, was associated with HBsAg levels. These results could provide a reference for developing a strategy for a functional cure for HBV.

## 2 Materials and methods

### 2.1 Donors and clinical information

The study protocol was reviewed as a multicenter study and approved by the local institutional ethics committee (National Institutes of Biomedical Innovation, Health and Nutrition, Osaka, Japan). All human experiments complied with the Declaration of Helsinki 1975, and all participants provided written informed consent. Clinical information, including the donor’s age, sex, blood HBsAg level, HBeAg, ALT, and HBV DNA levels, were collected at the hospitals (**Table S1**). PBMCs were isolated from 26 patients with CHB via density gradient centrifugation using either Ficoll-Paque Plus (Cytiva, Buckinghamshire, UK) or BD vacutainer CPT (Becton, Dickinson, and Co. [hereafter referred to as BD], Franklin Lakes, NJ, USA), according to their respective manufacturer’s instructions. PBMCs were immersed in fetal bovine serum (FBS) containing 10% dimethyl sulfoxide (DMSO) and stored in liquid nitrogen vapor until analysis.

### 2.2 Overlapping peptides

Overlapping peptides covering the HBs, HBcore, and HBpol regions were designed based on the consensus sequences covering genotypes A2, B2, C2, and D1 of HBV reported in Japanese patients (Details in **Supporting information**). A 15-mer peptide with a 9-mer overlapping with the surrounding sequences was designed for each antigen (**Table S2**).

### 2.3 Sorting, scRNA-seq library preparation, and sequencing

Frozen PBMCs were thawed, washed with RPMI1640 medium (Sigma-Aldrich, St. Louis, MO, USA) supplemented with 10% FBS (Sigma-Aldrich), 100 U/mL of penicillin, and 100 mg/mL streptomycin (Sigma-Aldrich; hereafter referred to as R10) and treated with 1 mL benzonase (50 U/mL; Merck, Darmstadt, Germany) in R10 for 30 min at 37°C. Cells were stained using a sorting panel (**Table S3**), and up to 20000 CD69^+^ non-naïve activated CD8^+^ T cells (defined by CD69/CD45RO/CD27) per donor were sorted into 20% FBS in phosphate-buffered saline (PBS). The frequency of cells located in the CD69-positive gate in isotype control staining samples was negligible with a level similar to that observed in the CD69-staining sample in the naïve CD8^+^ T cells (Tn) gate (**Figure S1**). Sorted cells (purity median = 95% [range 90–96%]) were centrifuged at 1000 ×*g* for 5 min, washed once with PBS, centrifuged at 2000 ×*g* for 5 min, resuspended in ~17 μL of PBS, and used as input for chromium 5′-kit (10× Genomics, Pleasanton, CA, USA) beads/donor. The scRNA-seq library was produced according to the manufacturer’s protocol. Libraries were combined and read using an Illumina NovaSeq 6000 platform (outsourced to AZENTA, Burlington, MA, USA).

### 2.4 scRNA-seq analysis

Raw sequence reads were analyzed using cellranger 6.1.2 (10× Genomics) with the human GRCh38 reference genome and the corresponding GTF file. The output matrices from cellranger were analyzed using *Seurat 4.0* (Details in **Supporting information**). Briefly, integrated Seurat objects were normalized and clustered. Differentially expressed genes (DEGs) were identified using *Seurat::FindMarkers*. Enriched biological processes were extracted using *clusterProfiler::enrichGO*. For gene set enrichment analysis (GSEA), genes used for the GO enrichment analysis were selected and biological processes were extracted using *clusterProfiler::gseGO*; the top two signatures were visualized using clusterProfiler::gseaplot. Module scores were calculated with the gene list from the GSEA molecular signature datasets, or selected genes presented in **Table S4**.

### 2.5 Long-term culture and flow cytometry

PBMCs were thawed, washed with R10, and treated with 1 mL benzonase (50 U/mL) in R10 for 30 min at 37°C. Cells were cultured for 10 days in the presence of 20 U/mL recombinant IL-2 (R&D systems) to expand antigen-specific CD8^+^ T cells, as previously described (2). After 10 days, the cells were restimulated with the corresponding peptides for 6 h, then stained by a panel of antibodies (**Table S5**) and analyzed using flow cytometry (Details in **Supporting information**).

Flow cytometry FCS files were analyzed using FlowJo (10.8.1; BD). Antigen-specific cells were denoted by their positivity for IFN-γ, TNFα, CD107A, or CD137. The frequency of HBV-specific CD8^+^ cells was determined by subtracting the value obtained via peptide-free stimulation (DMSO; background) from that obtained by each antigenic stimulation. After background subtraction, values ≤ 0.5% were considered negative (no response). Polyfunctionality was analyzed using Simplified Presentation of Incredibly Complex Evaluations (SPICE, version 6.1) (17).

### 2.6 Statistics

Statistical analyses were performed using R/Bioconductor (R 4.2.1/Bioconductor 3.16; **Table S6**), GraphPad Prism (8.3.0; GraphPad Software, San Diego, CA, USA) or SPICE). Groups were compared using the nonparametric Mann–Whitney *U* test. *P* < 0.05 was considered to indicate significance. For correlation analysis, correlation coefficients and *P*-values were calculated using Spearman’s correlation test. For the polyfunctionality analysis, *P*-values were calculated using SPICE software.

## 3 Results

### 3.1 Activated CD8^+^ T cells in patients with CHB using scRNA-seq

**Figure 1:**
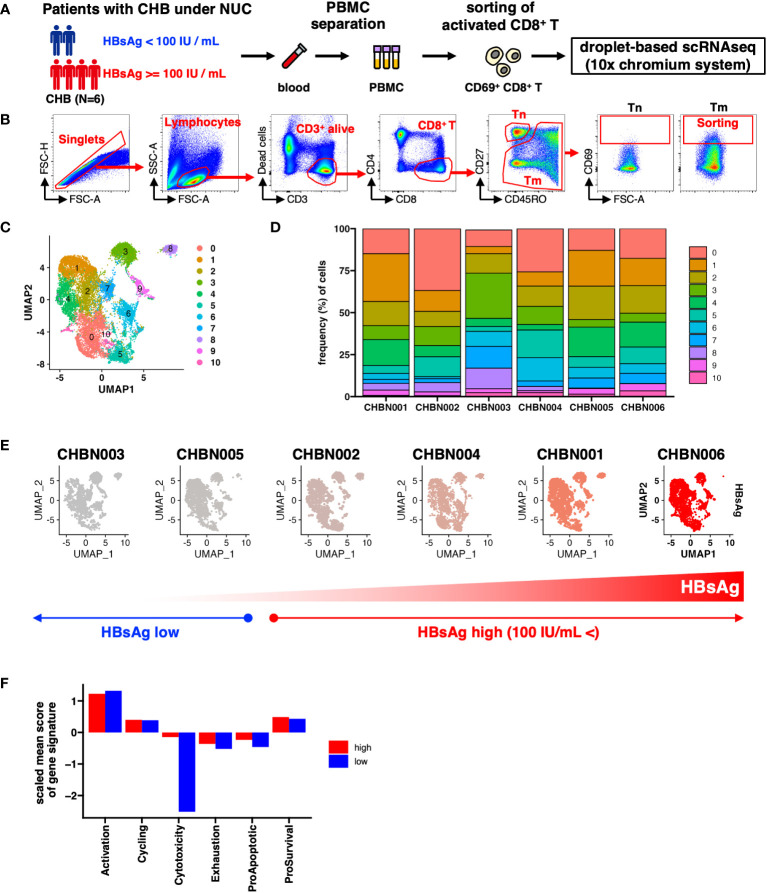
Single-cell gene expression profile of CD69^+^ activated CD8^+^ T cells in patients with chronic hepatitis B (CHB). **(A)** Schematic of the experimental workflow. **(B)** Gating strategy to monitor the transcriptional status of activated CD8^+^ T cells in patients with CHB. Tn: naïve T cells, Tm; memory T cells. **(C)** UMAP shows the clustering of CD69^+^ activated CD8^+^ T cells in the peripheral blood of patients with CHB. The numbers indicate the cluster number as defined using Seurat. **(D)** Relative cluster frequency in each patient. Colors indicate the cluster number as defined by Seurat. **(E)** UMAPs of each patient. The donor IDs are indicated as the headers, and the colors represent HBsAg levels in each donor. **(F)** The mean score of each gene signature in donors showing high or low HBsAg level is shown in the bar plot. The mean score is calculated as the average of each signature per cell per group.

To characterize CD8^+^ T cell subpopulations in patients with CHB undergoing NUC treatment, we conducted scRNA-seq analysis on activated CD8^+^ T cells (**Figures 1A, B**). 15230 cells were recovered from six donors, and the total cell pool was classified into 12 clusters (**Figure S2A**). Of these, Cluster 11 (CL11) had a very small number of (29 cells) and was detected only from certain donors; therefore, it was removed from subsequent analyses as noise (**Figures 1C, D**; **S2B–D**).

To determine the features of activated CD8^+^ T cells that associate with the HBsAg levels, we classified the patients into two groups: HBsAg-high (>100 IU/mL) and HBsAg-low (<100 IU/mL) (**Figure 1E**). We compared the six major functions of T cells in each patient group, based on the scores of their gene signatures: activation, cell cycle, cytotoxicity, exhaustion, and either proapoptotic or prosurvival gene signatures (**Table S4**). Cells with lower cytolytic scores were observed in the HBsAg-low group, suggesting that a relatively low cytolytic score was a more optimized signature for reduced HBsAg, independent of the cluster (**Figure 1F**).

**Figure 2:**
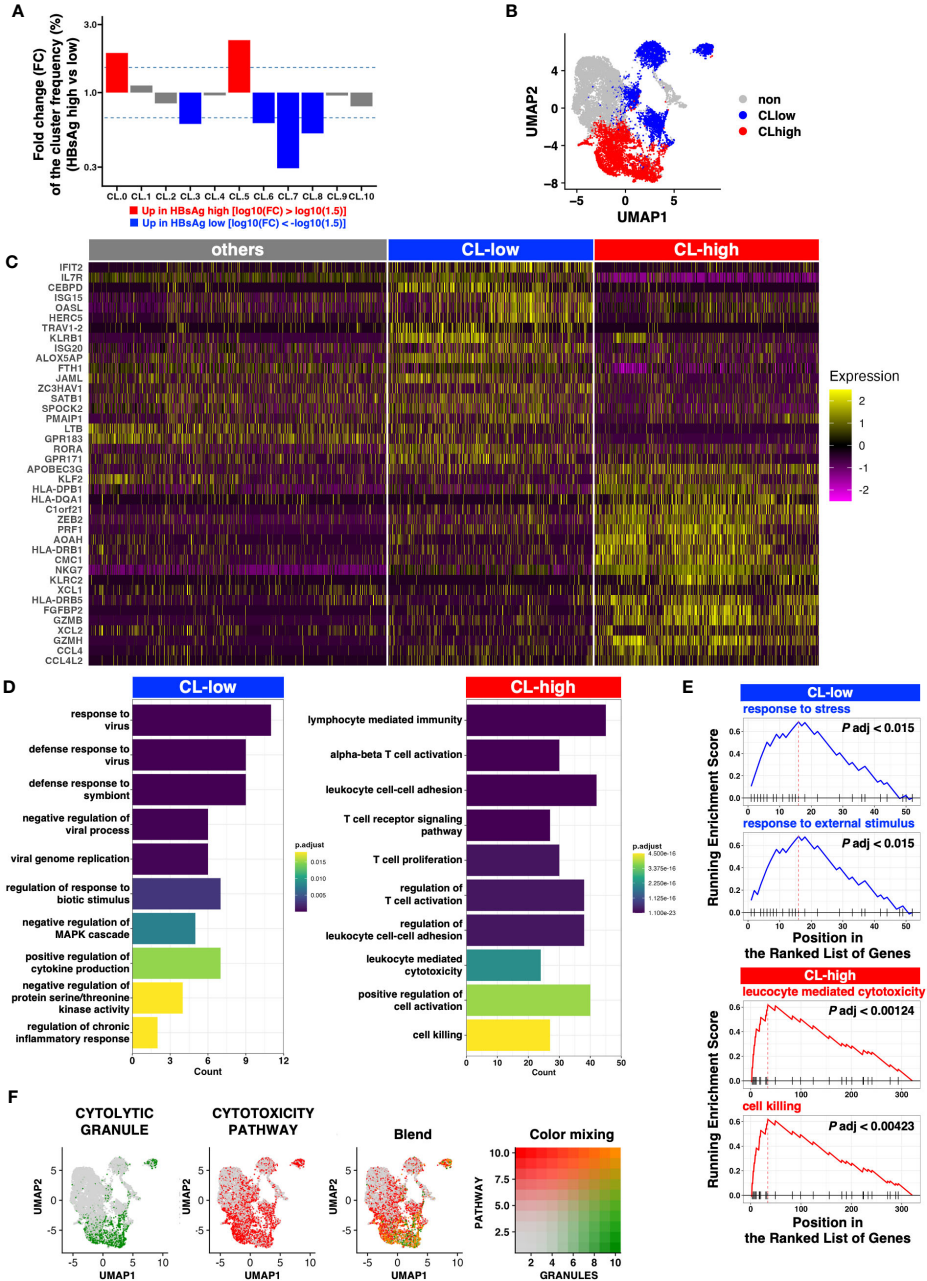
Clusters enriched in the HBsAg-high group exhibit a more cytolytic profile. **(A)** Fold change of the median frequency of cells in each cluster. **(B)** UMAP of the clusters enriched in the HBsAg-high (CL-high, red) and HBsAg-low (CL-low, blue) groups. **(C)** Heatmap indicating the markers highly expressed in the CL-low and CL-high groups. Top 20 DEGs from CL-low and CL-high groups were selected for the plot. **(D)** Bar plot indicating the enriched Gene Ontology (GO) pathways in each cluster (CL-low and CL-high). **(E)** Gene set enrichment analysis (GSEA) of CL-low and CL-high group gene signatures. Top two representative GSEA plots of each cluster group are shown. **(F)** Cytolytic module scores (signature genes for cytolytic granules and cytotoxicity pathways) depicted as a feature plot. Module scores of CYTOLYTIC GRANULE and CYTOTOXICITY PATHWAY are shown in green and red, respectively. The Blend panel indicates the blended plot of the two plots (left). Color mixing indicates the degree of color in the Blend panel.

Next, we explored the clusters biased to the HBsAg level. We examined the relative frequency of each cluster in each HBsAg level (**Figure S3**). Because of the limited number of samples, simple statistical comparisons only offer a weak indication without statistical significance. Even though, we found that HBsAg showed biased accumulations in several clusters. To highlight the differences, the fold changes of the median frequencies of each cluster were examined. The relative frequencies of CL0 and CL5 were higher than other CLs in the HBsAg-high group (**Figure 2A**), whereas those of CL3, CL6, CL7, and CL8 were higher in the HBsAg-low group. Therefore, we tentatively defined these characteristic clusters collectively as HBsAg-high clusters (“CL-high,” comprising CL0 and CL5) and the other clusters as HBsAg-low clusters (“CL-low,” comprising CL3, CL6, CL7, and CL8; **Figure 2B**).

To identify genes enriched in the CL-high and CL-low groups, we performed DEG analysis. Several cytolytic molecules, including *GZMA*, *GZMB* and *PRF1*, encoding granzyme A, granzyme B and perforin, respectively (18), were highly expressed in the CL-high group (**Figures 2C**, **S4A**). In addition, GZMH, GZMM, and GZMK are orphan granzymes (19) and the former two are highly expressed in clusters with high GZMB expression (CL0 and CL5). In addition, GZMK was highly expressed in CL0. (**Figure S4A**).

Several IFN-stimulated genes were highly expressed in the CL-low group (**Figures 2C**, **S3B**), but these characteristics were not identified from the cluster-wise comparison (**Figure S5**).

GO analysis revealed that the CL-low group genes were enriched in several antiviral pathways, but the CL-high group genes were enriched in cytolytic pathways exclusively (**Figure 2D**). This cytolytic bias of the CL-high group DEGs was further validated via GSEA (**Figure 2E**).

To comprehensively define cytotoxic activity, we examined the expression of genes involved in cytolytic granules and positive regulators of the T cell cytotoxic pathway as defined by MSigDB. When the module scores were calculated and plotted on UMAP, blended signatures were found to be enriched in the bottom-right half, particularly in CL0 and CL5 (**Figure 2F**).

In addition to lytic granules, the CL-high group tended to express genes encoding transcription factors involving either the exhaustion or effector functions of CD8^+^ T cells (e.g., TOX, NR4As, TNX21, or EOMES; **Figure S4C**). In contrast, CD127/IL7R expression was low in the CL-high group. CD127 is a marker of stem-like memory CD8^+^ T cells, which simultaneously express the central memory markers CCR7 and CD28 (**Figure S4D**). TOX expression is linked to HBV-specific CD8^+^ T cell dysfunction in chronic HBV infection (13), and CD127^+^ CD8^+^ T cells with stem cell-like properties, identified initially by the expression of TCF-1, are important for maintaining a robust CD8^+^ T cell response against chronic viral infections (20, 21). Hence, the CL-high group contained exhausted and dysfunctional CD8^+^ T cells, and the CL-low group contained more functional and long-lived memory-like CD8^+^ T cells with stem cell-like properties.

Taken together, these results suggest that among the CD69^+^ activated CD8^+^ T cells, which potentially harbor HBV-specific CD8^+^ T cells, higher HBsAg levels are correlated with higher cytotoxic activities, which may negatively affect HBsAg suppression.

### 3.2 Qualitative profiling of HBV-specific CD8^+^ T cells from patients with CHB

**Figure 3:**
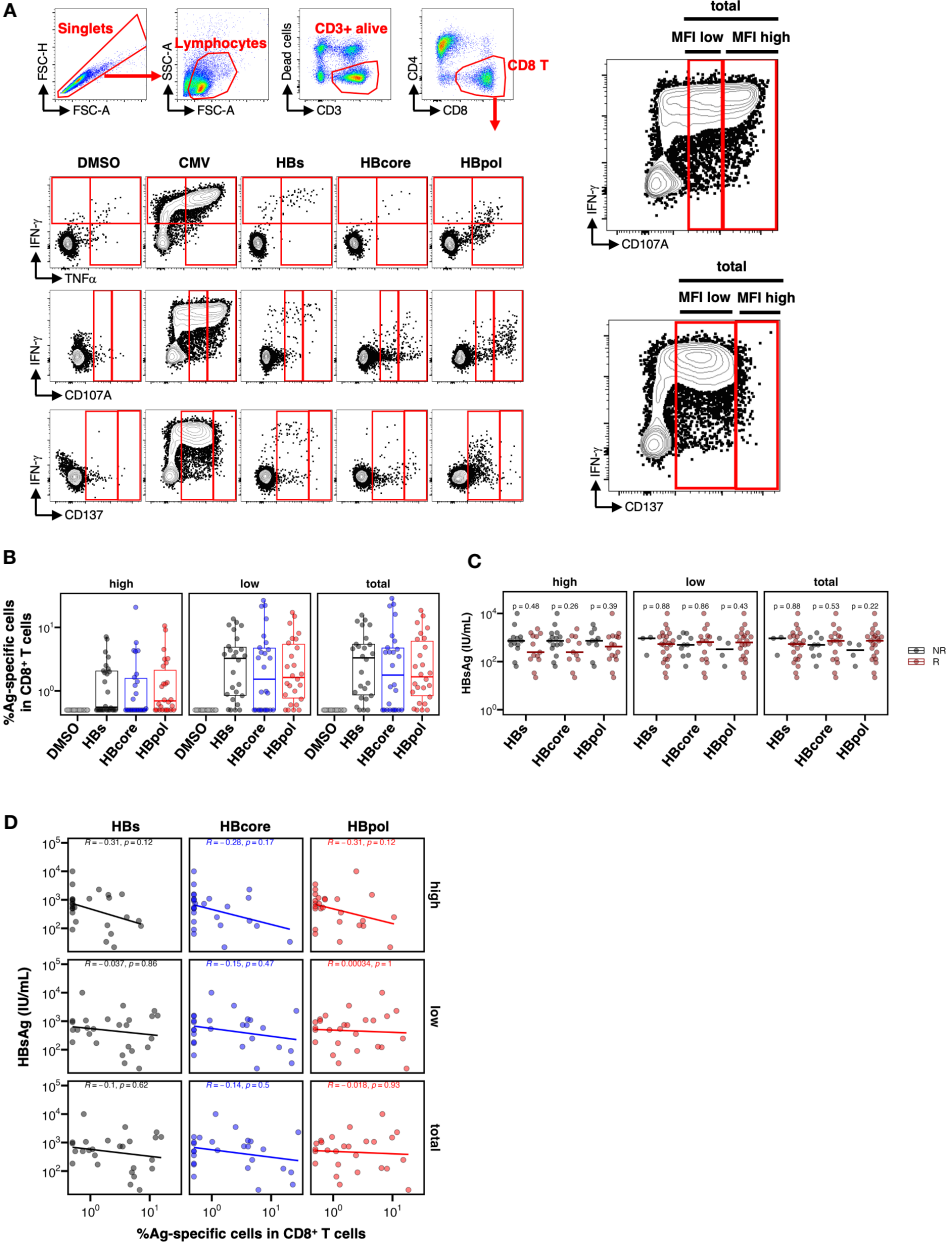
Low HBsAg levels are not associated with the overall pool of CD8^+^ T cells specific to either HBs-, HB-core, or HBpol. **(A)** Gating strategy for HBV-specific CD8^+^ T cells and representative flow cytometry plots of IFN-γ^+^, TNFα^+^, CD107A^+^, and CD137^+^ CD8^+^ T cells. For CD107A and CD137, three gating strategies (MFI high, MFI low, and total) were shown as enlarged image (right two images). **(B)** Frequency of HBV-specific CD8^+^ T cells stimulated by the indicated antigens (N=26); the peptide pool used for stimulation is shown on the X-axis. Three gating strategies: “MFI high”, “MFI low”, and “total” used to calculate the frequencies are labeled in the header as “high”, “low”, and “total” respectively. **(C)** Dotplots indicating the blood HBsAg levels in responders or non-responders, with the peptide pool used for stimulation shown on the X-axis. Cross bar indicates the median. **(D)** Scatter plots indicating the frequency of HBV-specific cells against the indicated antigen vs. the HBsAg level. R, responder; NR, non-responder. The mean difference between NR and R was examined using the Mann–Whitney *U* test. Correlations were examined using Spearman’s correlation analysis. Statistical non-significance is indicated by *P*-values.

Because our scRNA-seq analyses were based on bulk CD69^+^, and not HBV-specific, CD8^+^ T cells, we directly examined the association between the quantity and quality of HBV-specific CD8^+^ T cells via multi-parameter flow cytometry. PBMCs were cultured with overlapping peptide pools against each HB antigen (HBs, HBcore, or HBpol, **Figure S6**) for 10 days. HBV-specific CD8^+^ T cells were then detected via flow cytometry upon re-stimulation with the same antigen on the last day of staining for intracellular IFN-γ and TNFα and extracellular CD107A and CD137, which are considered markers of antiviral cytokine and cytotoxic activity, respectively (**Figure 3A**). Notably, to delineate cytolytic potential more comprehensively, for CD107A and CD137, we utilized three gating strategies (high, low, or total: **Figure 3A**; right) based on the mean fluorescent intensity (MFI) of each marker in response to CMVpp65. To maximize the detection of antigen-specific CD8^+^ T cells, cells expressing any of these four markers were defined as HBV-specific CD8^+^ T cells (**Figure 3B**). Accordingly, donors were classified as responders or non-responders based on the frequency of HBV-specific CD8^+^ T cells above the threshold (0.5% of alive CD8^+^ T cell subset). For instance, for MFI high gating (both CD107A and CD137 are high), HBV-specific CD8^+^ T cell responses were detected in 50%, 42%, and 62% of the 26 donors for HBs, HBcore, and HBpol, respectively (**Figure S7**). The number of responders was increased for MFI low or total gating (**Figure S7**).

We investigated the effect of HBV-specific CD8^+^ T cells on HBsAg levels and observed a trend toward lower HBsAg levels within the responder group to any HB antigen only in MFI high gating, although no significant difference was observed (HBs, *P*=0.48; HBcore, *P*=0.26; HBpol, *P*=0.39 in MFI high gating; **Figure 3C**). We further examined the association between the frequency of HBV-specific CD8^+^ T cells and HBsAg levels and found no correlation between HBs, HBcore, and HBpol-specific CD8^+^ T cells and HBsAg in any of three forms of gating (**Figure 3D**). Similarly, we analyzed the association of HBV-specific CD8^+^ T cells and ALT or HBV.DNA (**Figure S7**). Notably, the frequency of HBpol-specific CD8^+^ T cells in MFI high gating was correlated with the ALT level (**Figure S8C**).

### 3.3 Polyfunctional analysis reveals qualitative differences among HBs-, HBcore-, and HBpol-specific CD8^+^ T cells

To specify HBV-specific CD8^+^ T cells according to function, we separately analyzed each of the four markers used to define the total pool of HBV-specific CD8^+^ T cells (IFN-γ, TNFα, CD107A, and CD137). We confirmed that each of the four marker-positive CD8^+^ T cells could be classified separately as HBs, HBcore, or HBpol-specific CD8^+^ T cells (**Figure S9A**). However, correlation analyses of each marker showed no significant correlation between HBsAg and HBs-, HBcore-, or HBpol-specific CD8^+^ T cells (**Figure S9B**). These results suggest that a single parameter analysis is not sufficient to delineate the association between HBsAg levels and the quantity of HBV-specific CD8^+^ T cells.

**Figure 4:**
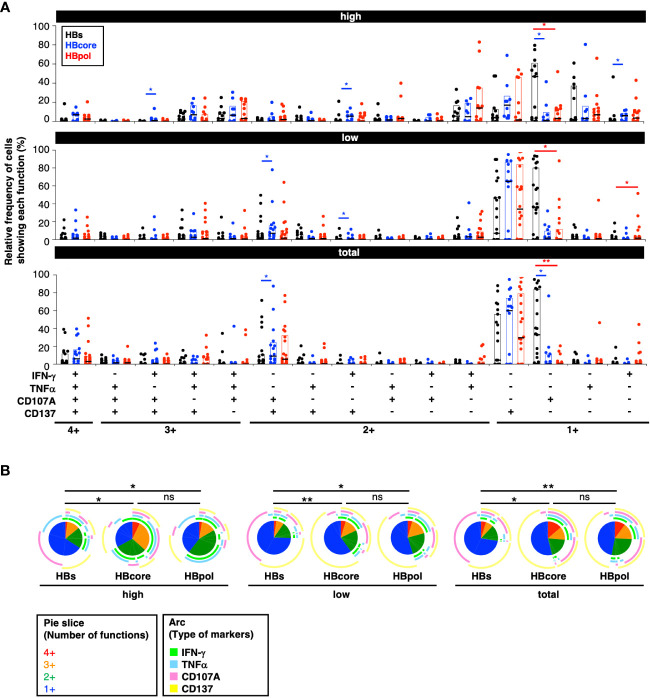
Quantitative differences in HBV-specific CD8^+^ T cells depending on the targeted antigen. **(A)** Relative frequency of HBV-specific CD8^+^ T cell subpopulations showing each function. *P*-values were calculated using the Wilcoxon matched-pair signed rank test and analyzed using SPICE software. Three gating strategies: “MFI high”, “MFI low”, and “total” used to calculate the frequencies are labeled in the header as “high”, “low”, and “total” respectively. **(B)** Frequencies of polyfunctional HBV-specific cells positive for each marker against the indicated antigens. *P*-values were calculated using the permutation test and analyzed using SPICE software. Statistical significance is indicated by asterisks based on *P*-values (*P<0.05, **P<0.01), ns, not significant.

Subsequently, we performed a multifunctional analysis to examine the qualitative aspects of each HBV-specific CD8^+^ T cell population. The CD107A single-positive subset for cytotoxic activity was significantly higher in HBs-specific CD8^+^ T cells than in HBcore-/HBpol-specific CD8^+^ T cells (**Figure 4A**). Overall, the percentage of monofunctional cells was the highest for HBs-specific CD8^+^ T cells and lowest for HBcore-specific CD8^+^ T cells (**Figure 4B**). Conversely, the percentage of most multifunctional cells (i.e., with the four markers) was highest in HBcore-specific CD8^+^ T cells and lowest in HBs-specific CD8^+^ T cells (**Figure 4B**). Furthermore, based on the classification of monofunctional cells, the percentage of cells positive for CD107A, a marker involved in cytotoxic activity, was extremely high in HBs-specific CD8^+^ T cells. However, the percentage of IFN-γ^+^ and TNFα^+^ cells, which are important for pathological control, was low (**Figure 4B**). These results suggest qualitative differences according to antigen specificity, with HBcore- and HBpol-specific CD8^+^ T cells being more multifunctional than HBs-specific CD8^+^ T cells (**Figure 4B**). Given that multifunctional cells positively affect disease control, our results might suggest that HBcore-specific CD8^+^ T cells are particularly important to reduce HBsAg levels.

### 3.4 The quantity of cytokine-producing HBcore-specific CD8^+^ T cells is inversely correlated with HBsAg level

**Figure 5:**
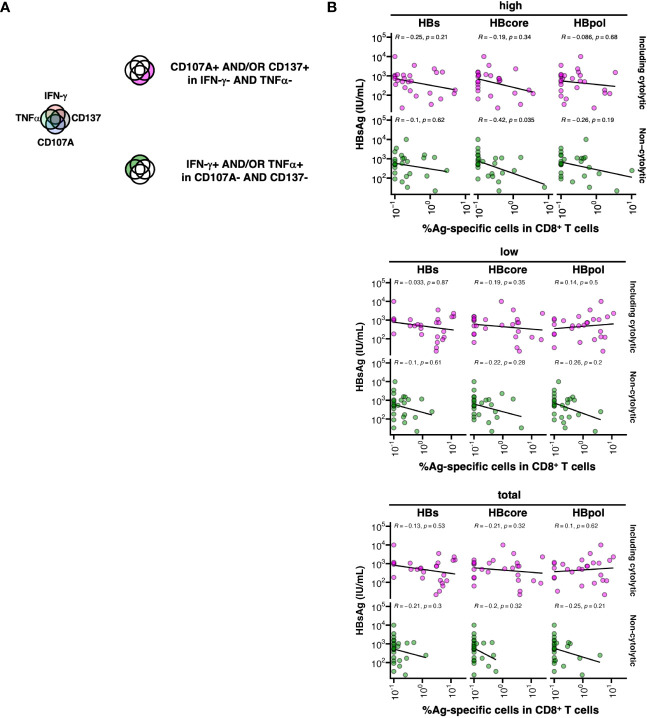
Association between low HBsAg levels and cytokine-producing HBcore and HBpol-specific CD8^+^ T cells. **(A)** Schematic of the grouping of HBV-specific CD8^+^ T cells into two subsets based on their cytolytic potentials. **(B)** The subset of HBV-specific CD8^+^ T cells against each antigen was defined by two marker sets (IFN-γ and TNFα or CD107a and CD137), and the correlation between the frequency of the subset (as depicted in the cartoon) and blood HBsAg levels was examined. The correlations were analyzed using Spearman’s correlation analysis. Statistical significance is indicated by *P*-values. Three gating strategies: “MFI high”, “MFI low”, and “total” used to calculate the frequencies are labeled in the header as “high”, “low”, and “total” respectively.

We examined the association between HBV-specific CD8^+^ T cell function and HBsAg levels. The cell populations were divided into two groups of their potential direct-lytic property depending on whether their cytokine nature (IFN-γ and TNFα) was expressed. Specifically, “including cytolytic” HBV-specific CD8^+^ T cells were defined as CD107A^+^ and/or CD137^+^ and IFN-γ^-^ and TNFα^-^, while “non-cytolytic” HBV-specific CD8^+^ T cells were defined as IFN-γ^+^ and/or TNFα^+^ and CD107A^-^ and CD137^-^ (**Figure 5A**). Among the HBcore-specific CD8^+^ T cells, the frequency of “non-cytolytic” cells defined by MFI high gating was significantly inversely correlated with HBsAg levels (**Figure 5B**, R=-0.42, *P*=0.035). In contrast, among the HBs- and HBpol-specific CD8^+^ T cells, cells were neither classified as “including cytolytic” nor “non-cytolytic”, and no correlation with HBsAg levels was observed. These results suggest that among the HBcore-specific CD8^+^ T cells, cells with particularly “non-cytolytic” characteristics potentially contribute to HBsAg control under NUC treatment.

## 4 Discussion

We investigated the features of HBV-specific CD8^+^ T cells in the PBMCs of patients with CHB, with particular emphasis on their cytolytic potential. scRNA-seq analysis of activated CD8^+^ T cells revealed a possible negative effect of the cytotoxic profile of these cells on HBsAg level reduction. There are numerous activated CD8^+^ T cells with cytolytic properties expressing CD38, but not CD69, in human PBMC; therefore, we may not capture the entire pool of activated CD8^+^ T cells completely. However, our finding was subsequently supported by the result of a multifunctional analysis of HBV-specific CD8^+^ T cells.

We defined a subpopulation of HBV-specific CD8^+^ T cells with weak cytolytic capacity based on IFN-γ and/or TNFα-positivity but CD107A and CD137 negativity after *in vitro* TCR stimulation. The frequency of this subpopulation in HBcore-specific, but not HBs- or HBpol-specific CD8^+^ T cells, was inversely correlated with HBsAg levels. In contrast, no association was found between HBsAg and each HBV-specific CD8^+^ T cells that were positive for CD107 and/or CD137, which were considered to have high cytolytic capacity. These results might suggest two important aspects concerning the reduction in HBsAg levels (1): the qualitative differences in HBV-specific CD8^+^ T cell function, and (2) the selection of antigen specificity of CD8^+^ T cells.

The pros and cons of cytotoxic activity, especially in HBV-specific CD8^+^ T cells, have long been debated in terms of HBV control. Studies on mouse models of HBV pathogenesis have shown that CD8^+^ T cells initially induce hepatocyte apoptosis rapidly via both perforin- and Fas-dependent pathways and kill HBV-expressing hepatocytes (22, 23). The non-cytolytic control of HBV, which is mediated by the release of antiviral cytokines such as IFN-γ and TNFα, can potentially act on multiple HBV-infected hepatocytes without exacerbating liver damage. The anti-HBV effect of T cells via IFN-γ and TNFα has been observed in transgenic HBV mouse models (24) and is effective against human hepatocytes that replicate HBV (14). An APOBEC3-mediated pathway involving T cells with non-cytotoxic activity has also been reported (25). However, the significance of the cytotoxic activity of HBV-specific CD8^+^ T cells in patients undergoing NUC therapy remains unclear.

Studies on HBs-transgenic mice have shown that HBV replication is inhibited by HBs-specific CD8^+^ T cells, even though HBs-specific CD8^+^ T cells have no harmful activity on infected cells (26). HBs-specific CD8^+^ T cells kill only a small fraction of HBV-positive hepatocytes, suggesting their cytotoxic activity not sufficient for virus elimination (27). Conversely, a cytotoxic activity-independent IFN-γ production-dependent contribution to suppressing HBV replication has been suggested. For instance, HBV-specific CD8^+^ T cells are involved in the IFN-γ- and TNFα-production-dependent control of HBV replication and cccDNA reduction without direct contact with target cells (14, 28, 29).

To reveal the functional profile of CD8^+^ T cells involving HBV control, most studies have used bulk CD8^+^ T cells. Through transcriptomic analysis of CD8^+^ T cells of patients under NUC therapy with the frequency of HBV-specific CD8^+^ T cells defined using ELISPOT, Rivino et al. showed that *PD-1* mRNA expression in bulk CD8^+^ T cells is upregulated in the non-relapse group, but the relationship of cytotoxic activity was not examined in detail (2). In their study of a large cohort of 243 patients, Le Bert et al. conducted a CyTOF analysis of PBMCs with different serum HBsAg levels and found no correlation with HBsAg levels in bulk CD8^+^ T cells (15).

Early data show that an epitope against HBcore, HBcore18-27, is associated with the spontaneous resolution of HBV infection in HLA-A2-positive patients (8, 30). Cheng et al. performed a comprehensive HBV-specific CD8^+^ T cell analysis using a multiplex-pMHC tetramer library restricted to HLA-A*11:01 in three different stages of CHB patient groups (31). Although the overall magnitude of antigen specificity did not differ among the patient groups, HBcore- and HBpol-specific CD8^+^ T cells were more frequent than did HBs- and HBx-specific CD8^+^ T cells in each group. Consistent with our results, a higher frequency of HBcore169-specific CD8^+^ T cells was associated with more optimized disease control. However, most of these studies have been limited because of the greater focus on specific HLA-binding epitopes.

In our assays, we used overlapping peptides corresponding to HBV antigens to cover all HLA types. To minimize the culture effect, we have tried to detect *ex vivo* HBV-specific CD8^+^ T cells without culture in a small number of donors (N=8) and examined the frequency of HBV-specific CD8^+^ T cells and proportion of responders by comparing the result of the long-term culture method. However, the very few dots in the FACS plot were detected by *ex vivo* analysis (**Figures S11A**, **S11B**). Consequently, the number of responders was quite low (**Figure S11C**). Therefore, we focused on the culture method. Furthermore, because we wanted to detect antigen-specific CD8^+^ T responses independent of donor HLA, the use of multimer was not an option.

Le Bert et al. used a similar analytical approach to identify HBV-specific T cells using overlapping peptides (15). They found that the number, but not the function, of HBs-specific T cells was inversely correlated with serum HBsAg levels. Although not significant, the number of HBcore-specific T cells tended to be inversely correlated with HBsAg levels. These results are mostly consistent with our results, although the authors used the entire T cell population for their ELISPOT analysis, including CD8^+^ T cells and CD4^+^ T cells. Moreover, the frequency of HBV-specific CD8^+^ T cells was higher in patients with hypoviremic CHB, and HBcore-specific and HBpol-specific CD8^+^ T cells were usually easier to detect than HBs-specific CD8^+^ T cells (2, 10, 32). In our flow cytometry assays, HBcore-specific CD8^+^ T cells were the most easily detected. Mason et al. found no significant difference in the frequency of responders to each antigen (33), consistent with our results. In a recent cross-sectional study of children and adults with chronic HBV, a longer duration of HBsAg exposure was found to be correlated with a lower frequency of HBs-specific T cells (15). Although age should be considered, the limited number of samples in this study does not exclude this effect. Recently, Rivino et al. reported the functional profiles of both antigen-specific T cells and global non-antigen-specific immunity in patients with CHB during and after discontinuation of NUC therapy and determined the number of HBV-specific T cells amplified *in vitro* during NUC therapy (2). CD8^+^ T cell responses were significantly higher in patients who did not relapse after NUC therapy, with a significantly higher frequency of HBcore- and HBpol-specific responses at different time points. These data suggest that the detection of proliferative HBcore- and HBpol-specific T cells *in vitro* during NUC therapy can be used to predict whether a patient can safely discontinue NUC therapy. Moreover, phenotypic differences have been observed between HBcore and HBpol. *Ex vivo* analysis using pMHC multimers revealed that HBcore-specific T cells are the most abundant *ex vivo*, have a phenotype consistent with T effector memory cells, and proliferate most efficiently *in vitro* (10, 32). Conversely, HBpol-specific T cells are more heterogeneous, exhibit a partly naïve phenotype, are less proliferative *in vitro*, and may be more exhausted, as defined by the high expression of KLRG1, TOX, and others. Therefore, it may be more difficult to restore HBpol function than HBcore function.

We did not directly evaluate perforin or granzyme, which are direct indicators of cytolytic capacity, in HBV-specific CD8^+^ T cells. However, as granzyme has a non-lytic function (34), we selected CD107A and CD137 as more convenient and unambiguous indicators of cytolytic capacity after *in vitro* stimulation.

Of note, throughout the study, we demonstrated the association, but not causal relationship, between the characteristics of T cells and HBsAg level. Thus, the association maybe indirect or even inverse (e.g. high HBsAg levels may affect the characteristics of T cells).

In summary, our findings suggest that the selective induction of IFN-γ and/or TNFα-producing HBcore-specific CD8^+^ T cells with weak cytolytic capacity among HBV-specific CD8^+^ T cells may lead to reduced HBsAg levels. One of the limitations of this study is that we only analyzed PBMCs, and not liver cells. A CD8^+^ T cell subpopulation that contributes to HBV elimination in the liver has to be clarified in further analyses. Moreover, the mechanism underlying the antigen-dependent induction of qualitatively different CD8^+^ T cells remains unclear. We expect our findings to be used as a basis to evaluate the efficacy of immunotherapy aimed at a functional cure. In current therapeutic vaccine development, multiple antigens, including HBcore, rather than HBs alone, are used (35). Therefore, our approach can be used as an evaluation method for the development of new immunotherapies and can hold importance in future vaccine development strategies.

## Data availability statement

The datasets presented in this study can be found in online repositories. The name of the repository and accession number can be found here: ENA - PRJEB66332.

## Ethics statement

The studies involving humans were approved by the local institutional ethics committee (National Institutes of Biomedical Innovation, Health and Nutrition, Osaka, Japan). The studies were conducted in accordance with the local legislation and institutional requirements. The participants provided their written informed consent to participate in this study.

## Author contributions

ST: Methodology, Visualization, Writing – original draft, Conceptualization, Formal Analysis, Funding acquisition, Investigation. SY: Resources, Writing – review & editing, Supervision, Conceptualization. YM: Investigation, Writing – review & editing. HM: Investigation, Writing – review & editing. RS: Resources, Writing – review & editing. SK: Resources, Writing – review & editing. TH: Resources, Writing – review & editing. MM: Resources, Writing – review & editing. MS: Resources, Software, Writing – review & editing. MK: Resources, Writing – review & editing. YA: Resources, Writing – review & editing. TT: Resources, Writing – review & editing. VA: Supervision, Writing – original draft. TK: Resources, Supervision, Conceptualization, Writing – review & editing. TY: Funding acquisition, Supervision, Writing – original draft, Conceptualization, Investigation, Project administration.
