# Supplementary figures and images for: Hepatitis B surface antigen reduction is associated with hepatitis B core-specific CD8+ T cell quality
Source: Front Immunol. 2023 Oct 18;14:1257113. doi: 10.3389/fimmu.2023.1257113 (PMC10619684; doi:10.3389/fimmu.2023.1257113)

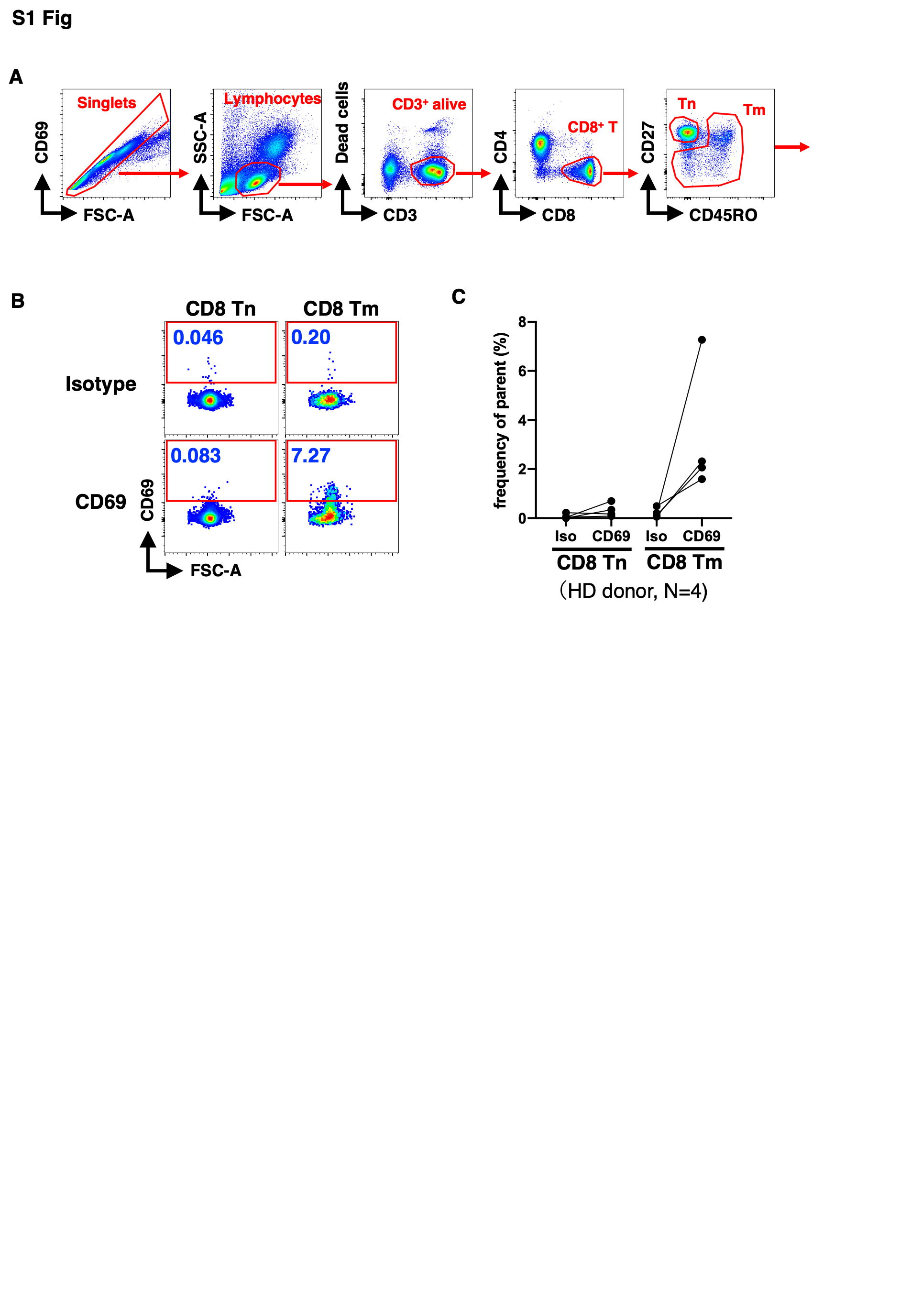

Supplement: Supplementary file 1 [file DataSheet_1.zip › Supplementary Figure 1.TIFF]

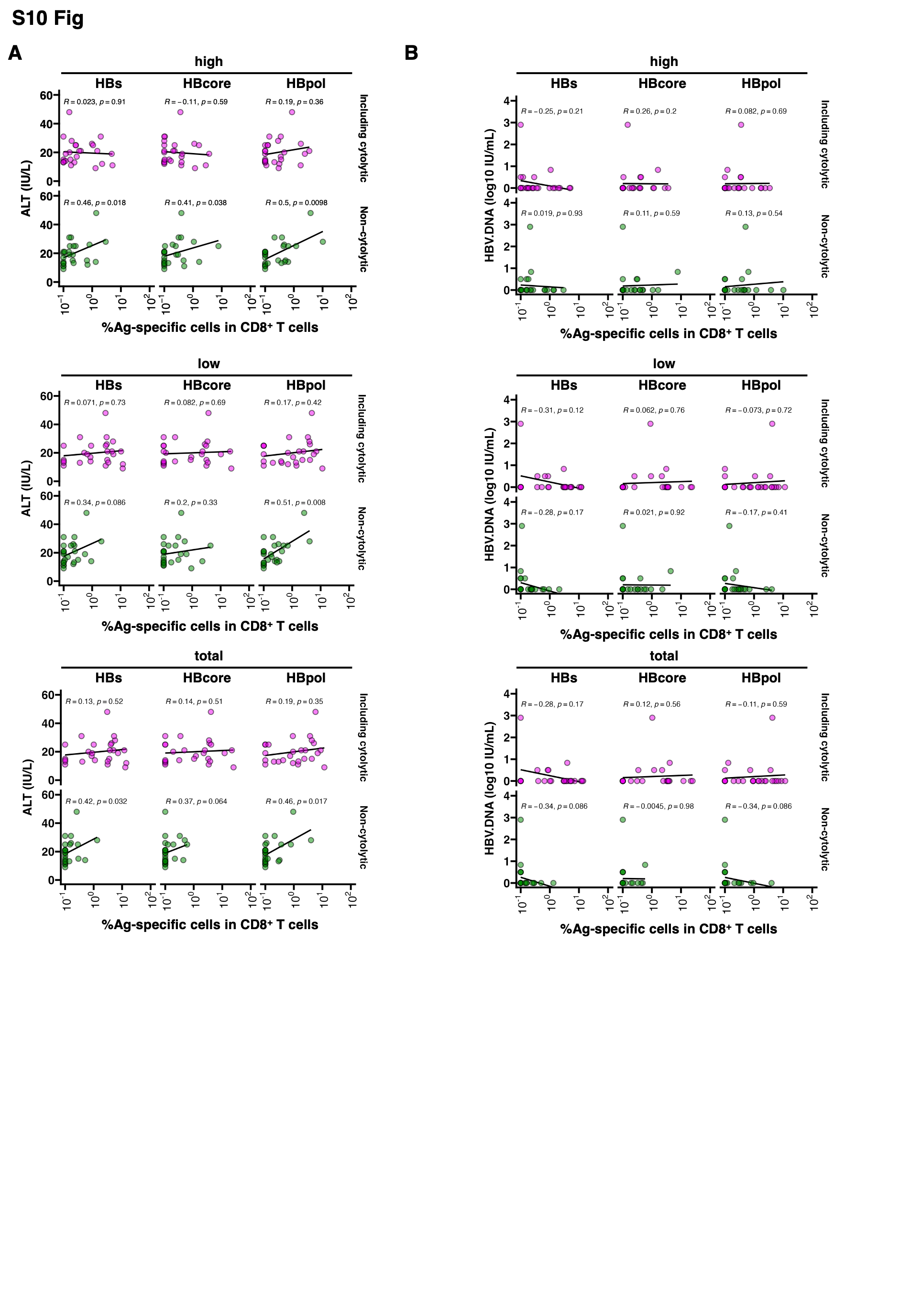

Supplement: Supplementary file 1 [file DataSheet_1.zip › Supplementary Figure 10.TIFF]

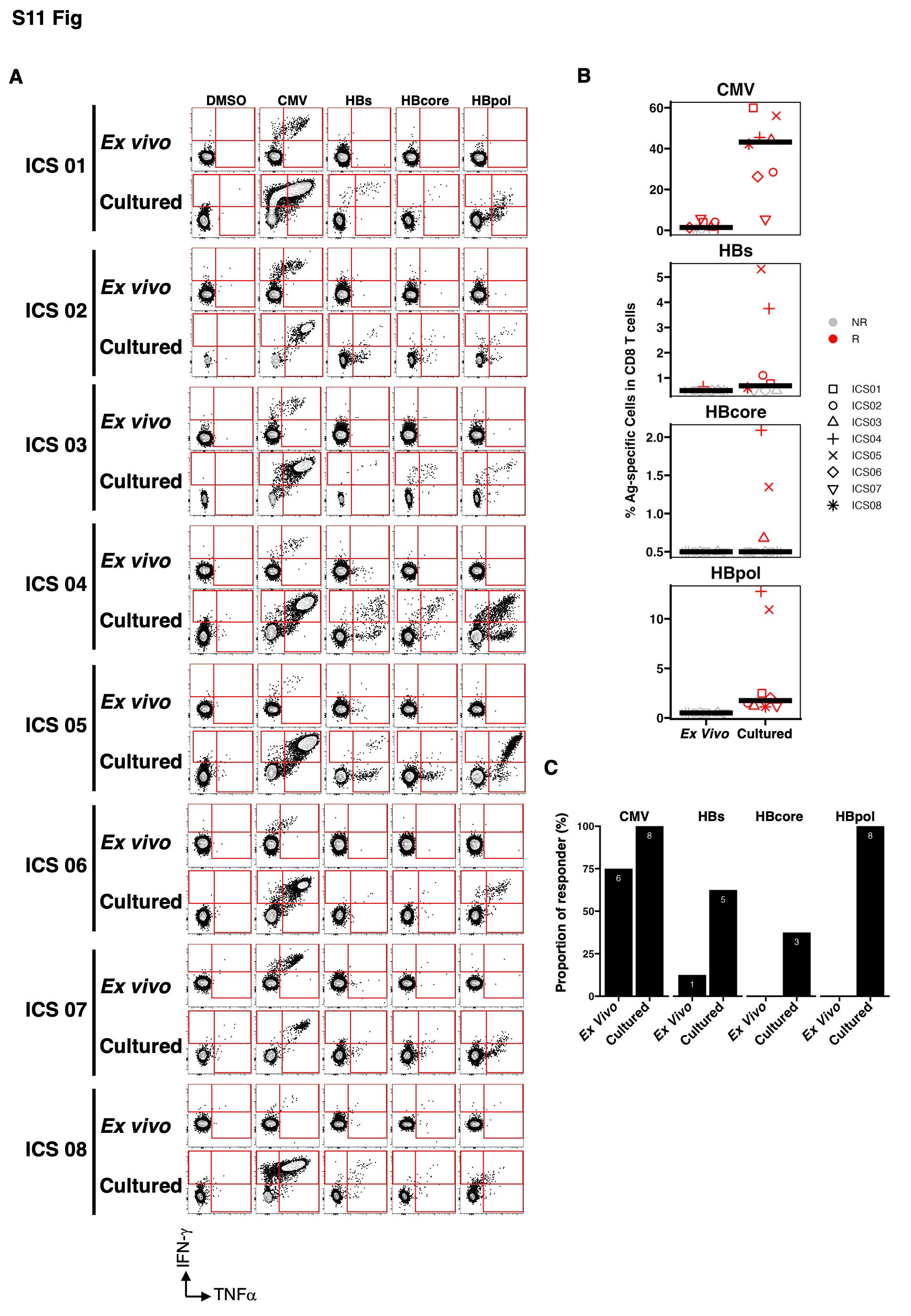

Supplement: Supplementary file 1 [file DataSheet_1.zip › Supplementary Figure 11.TIFF]

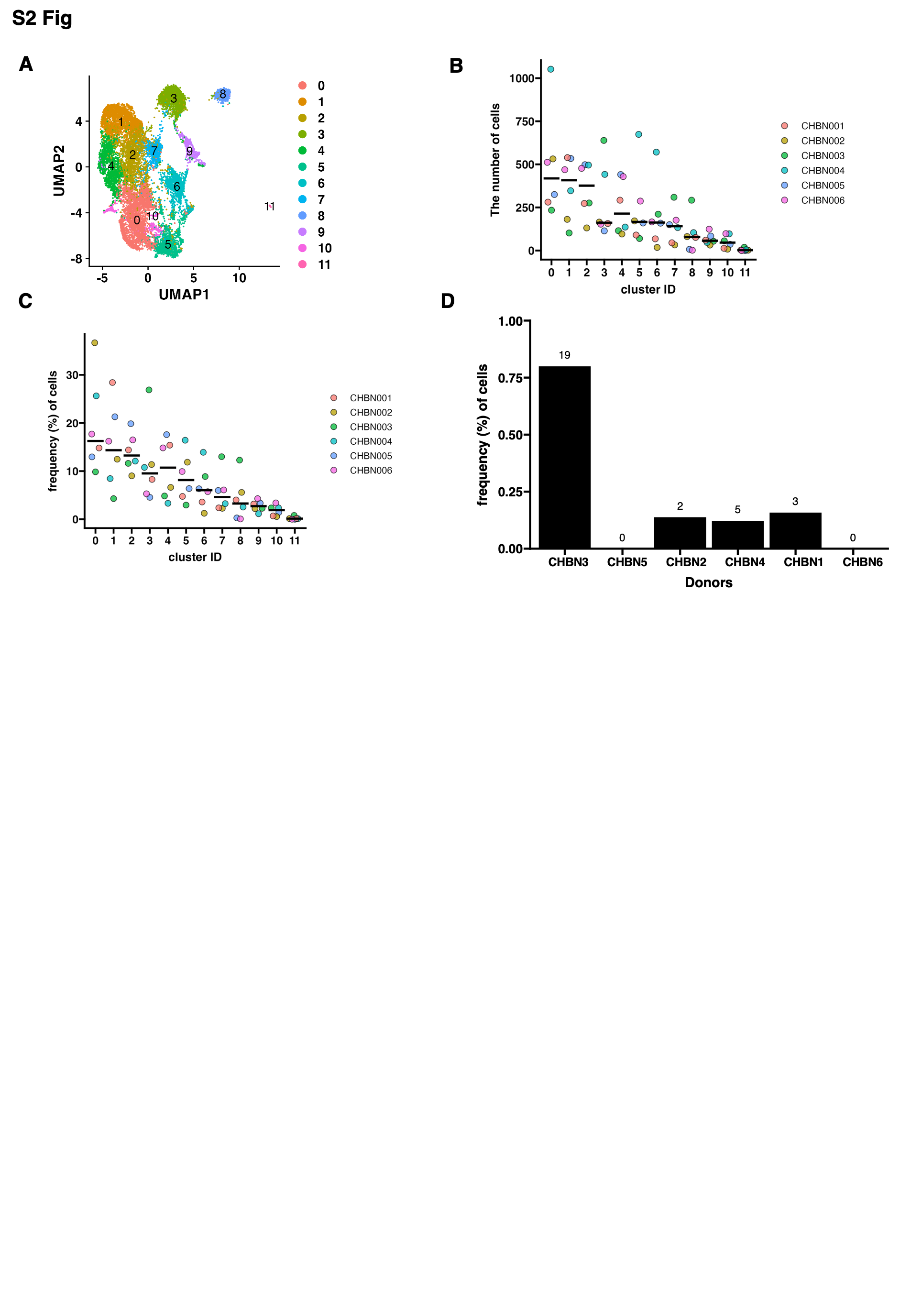

Supplement: Supplementary file 1 [file DataSheet_1.zip › Supplementary Figure 2.TIFF]

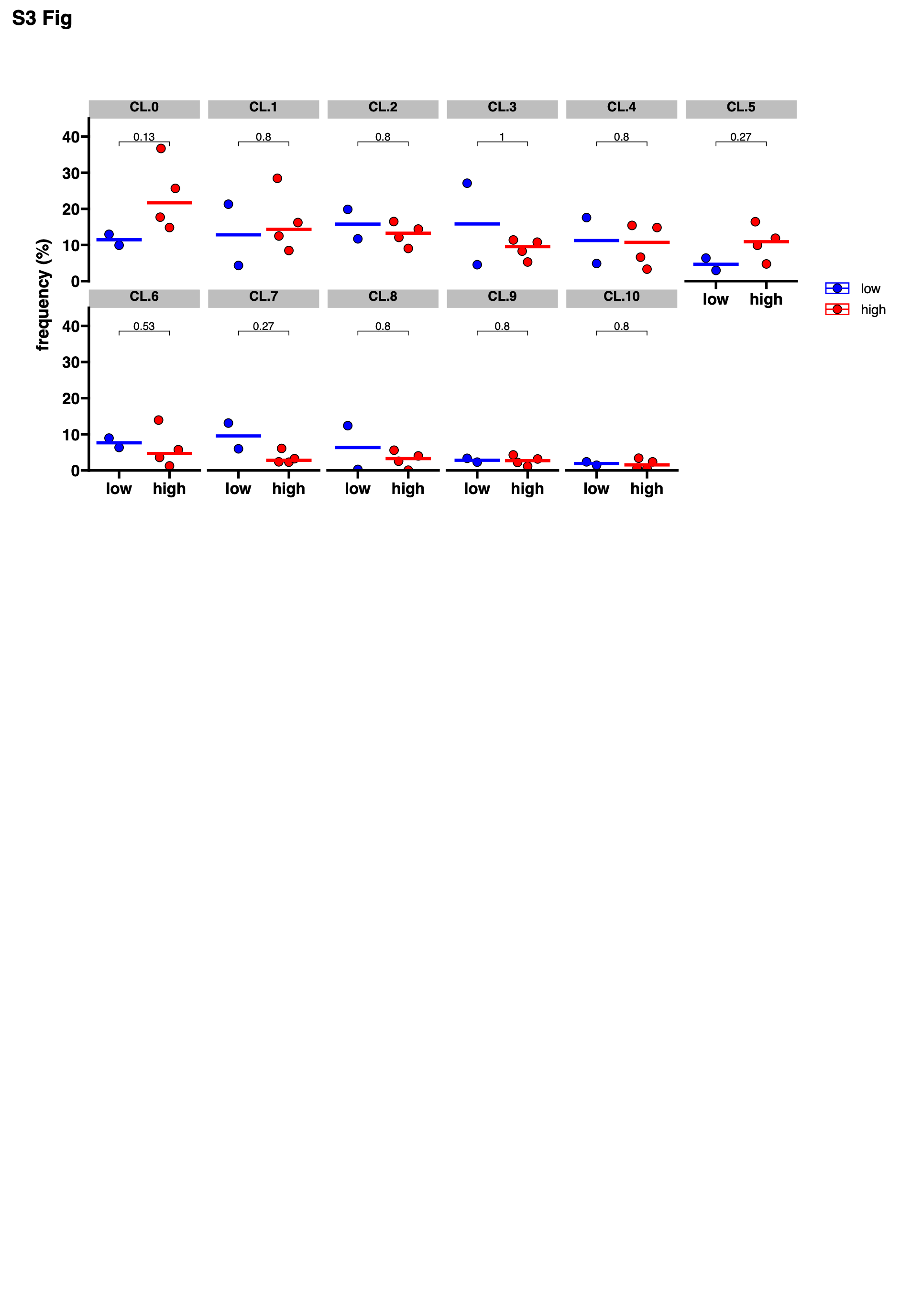

Supplement: Supplementary file 1 [file DataSheet_1.zip › Supplementary Figure 3.TIFF]

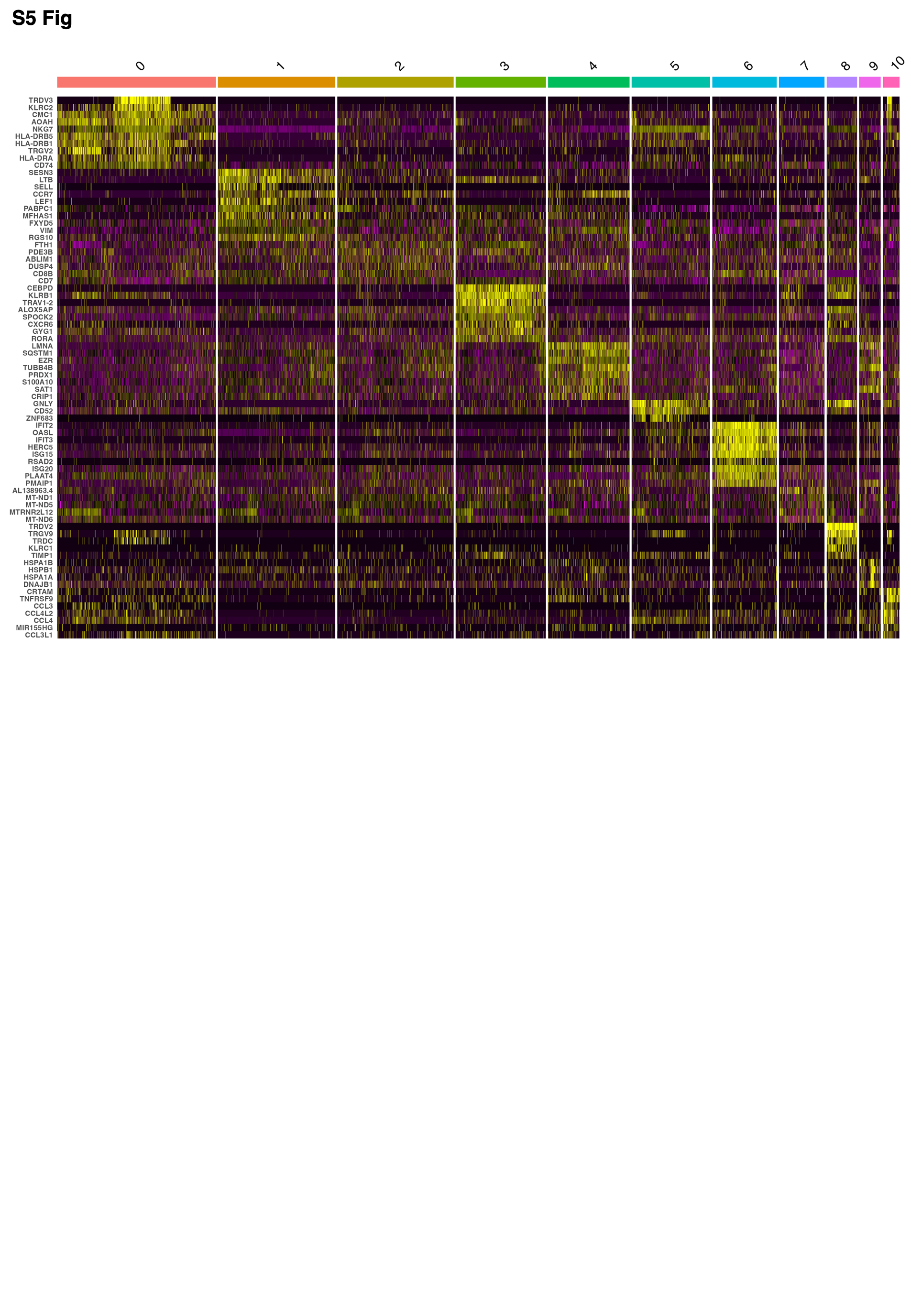

Supplement: Supplementary file 1 [file DataSheet_1.zip › Supplementary Figure 5.TIFF]

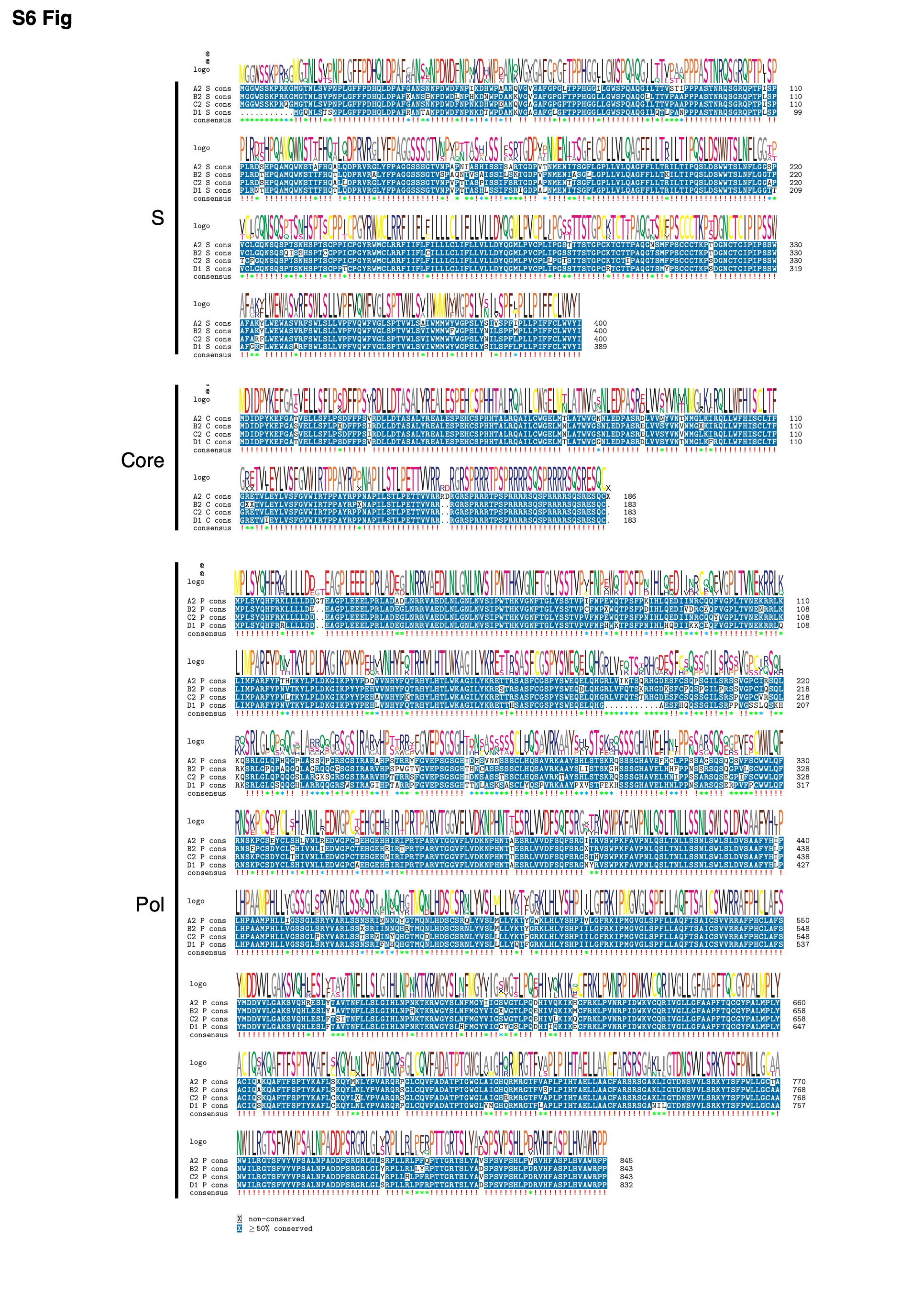

Supplement: Supplementary file 1 [file DataSheet_1.zip › Supplementary Figure 6.TIFF]

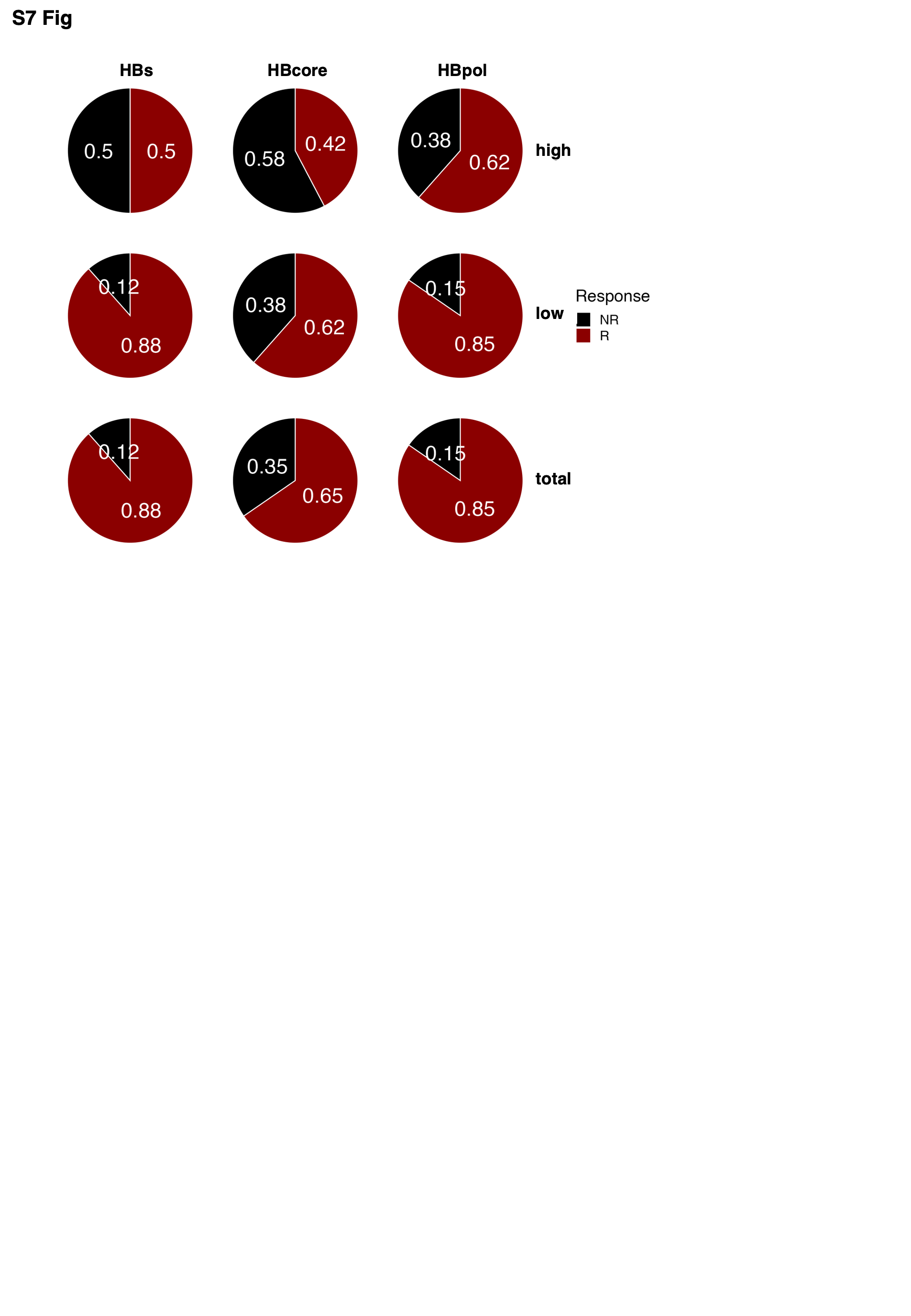

Supplement: Supplementary file 1 [file DataSheet_1.zip › Supplementary Figure 7.TIFF]

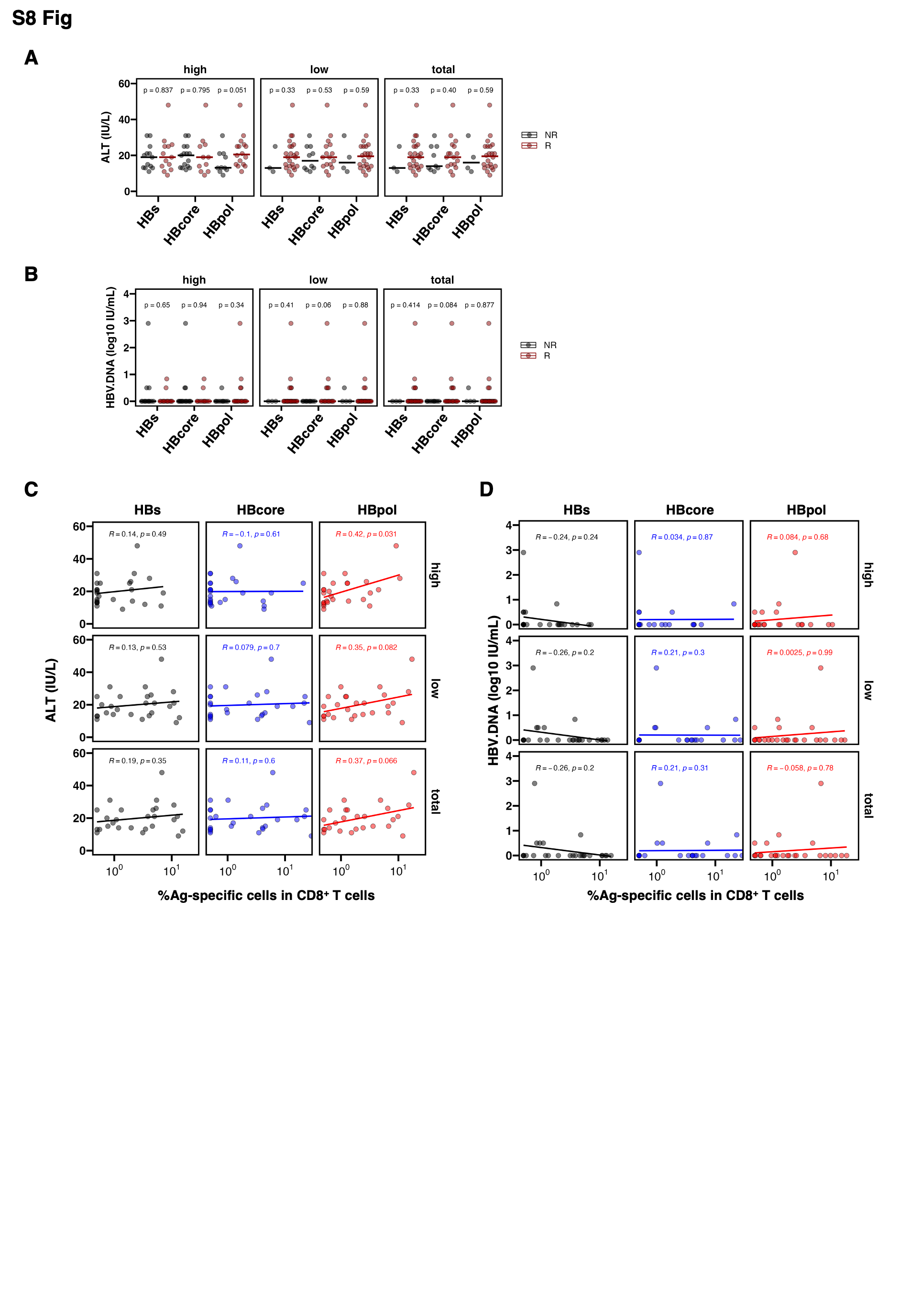

Supplement: Supplementary file 1 [file DataSheet_1.zip › Supplementary Figure 8.TIFF]

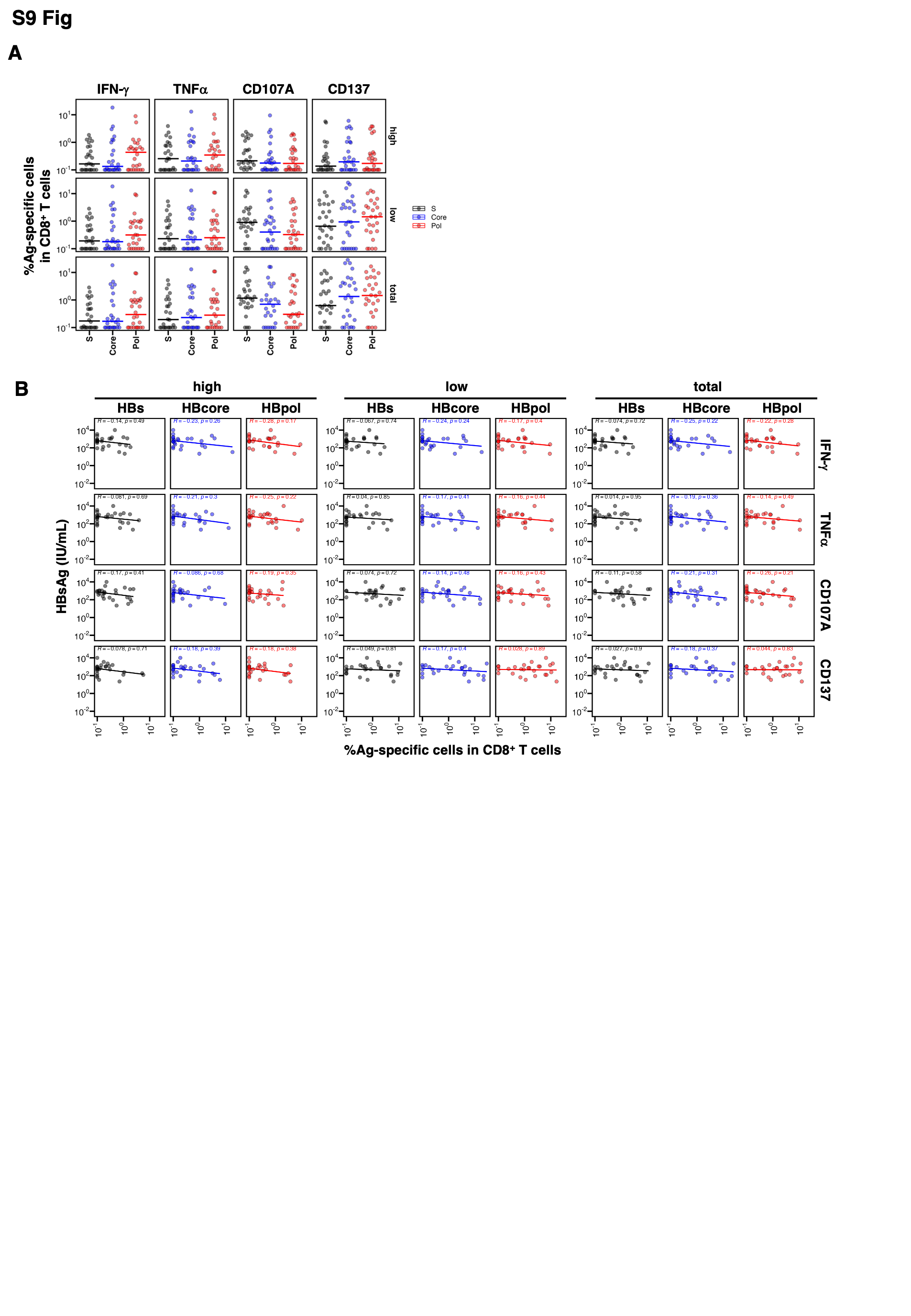

Supplement: Supplementary file 1 [file DataSheet_1.zip › Supplementary Figure 9A_B.TIFF]

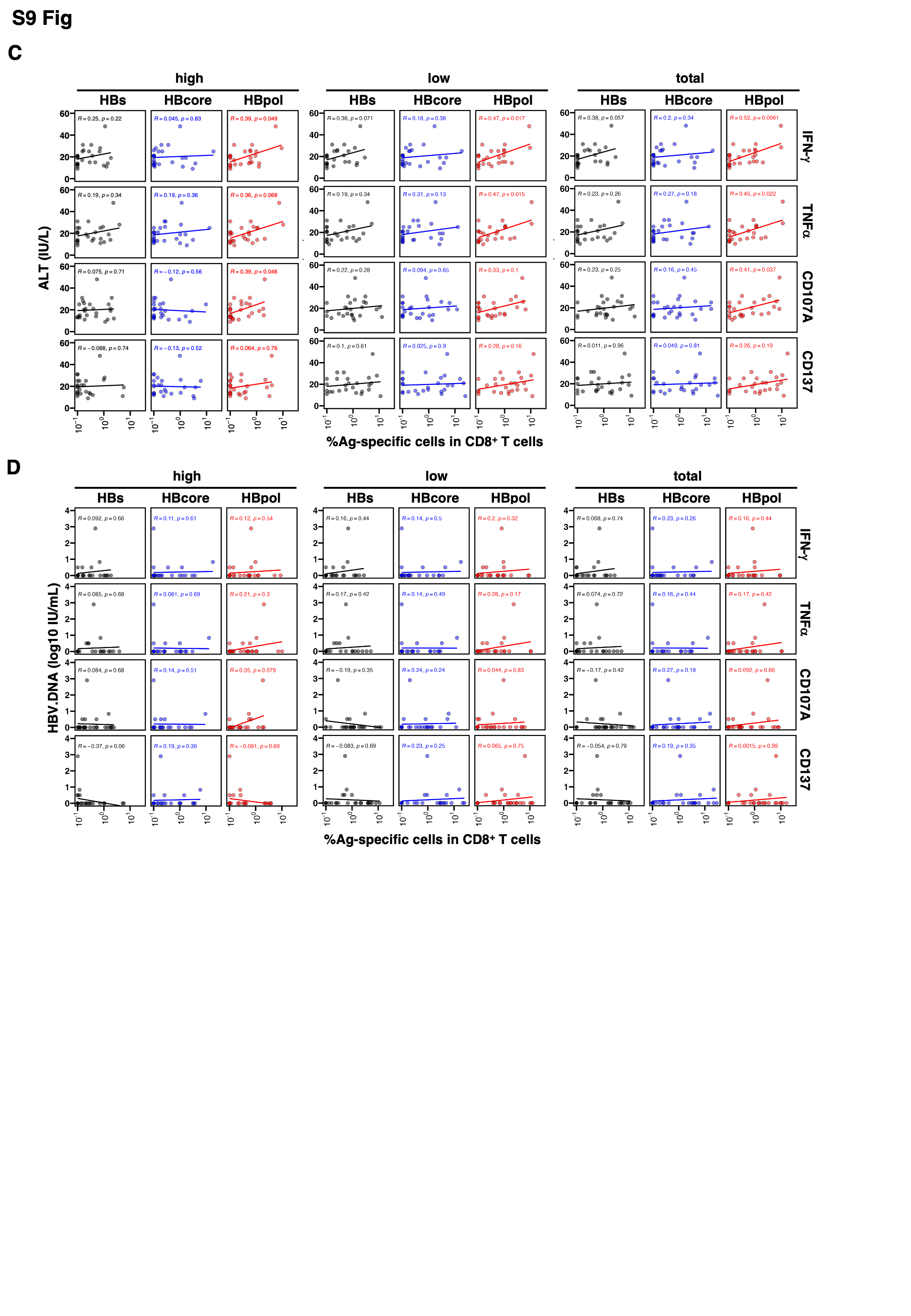

Supplement: Supplementary file 1 [file DataSheet_1.zip › Supplementary Figure 9C_D.TIFF]

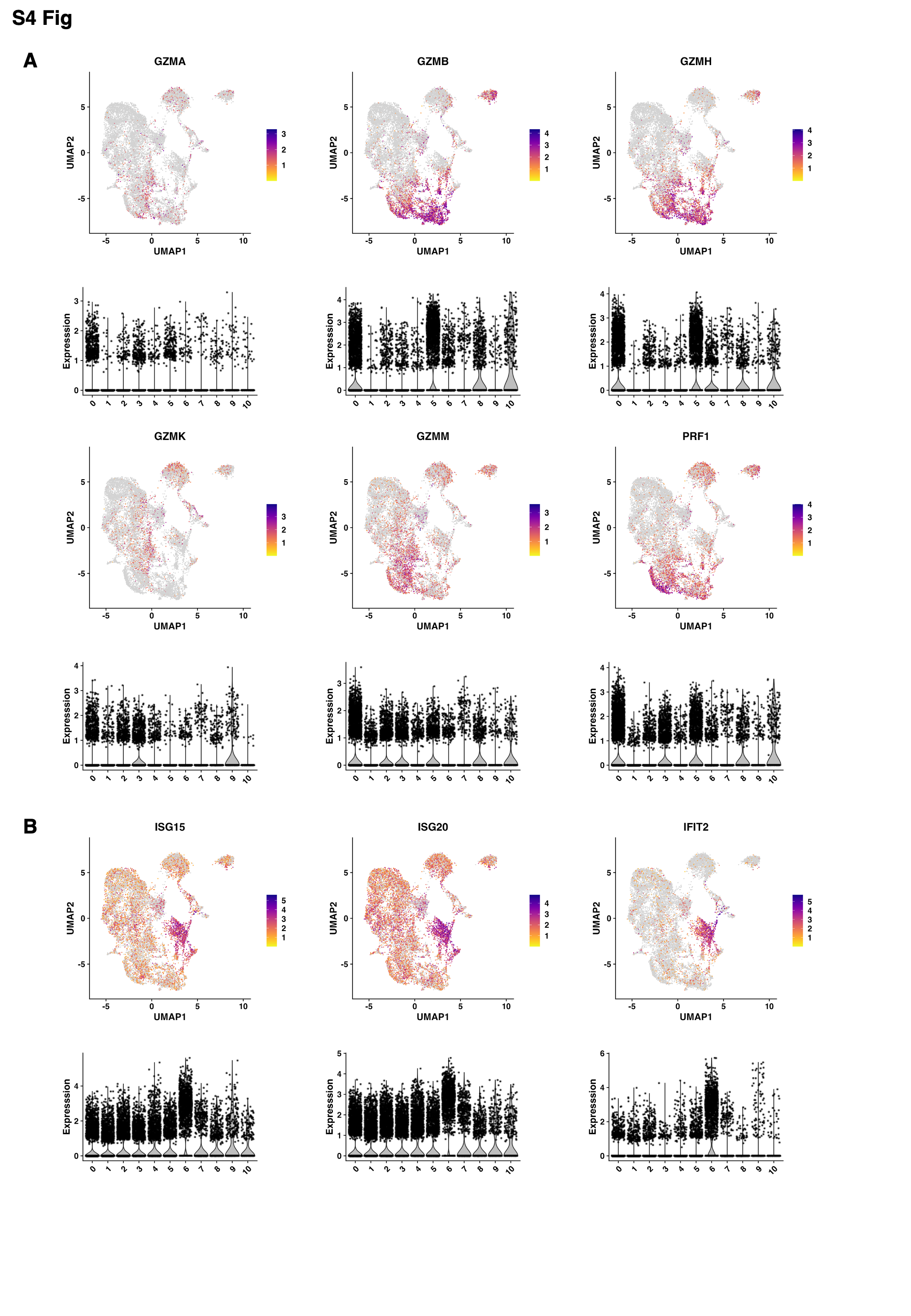

Supplement: Supplementary file 1 [file DataSheet_1.zip › Supplementary Figures 4A_B.TIFF]

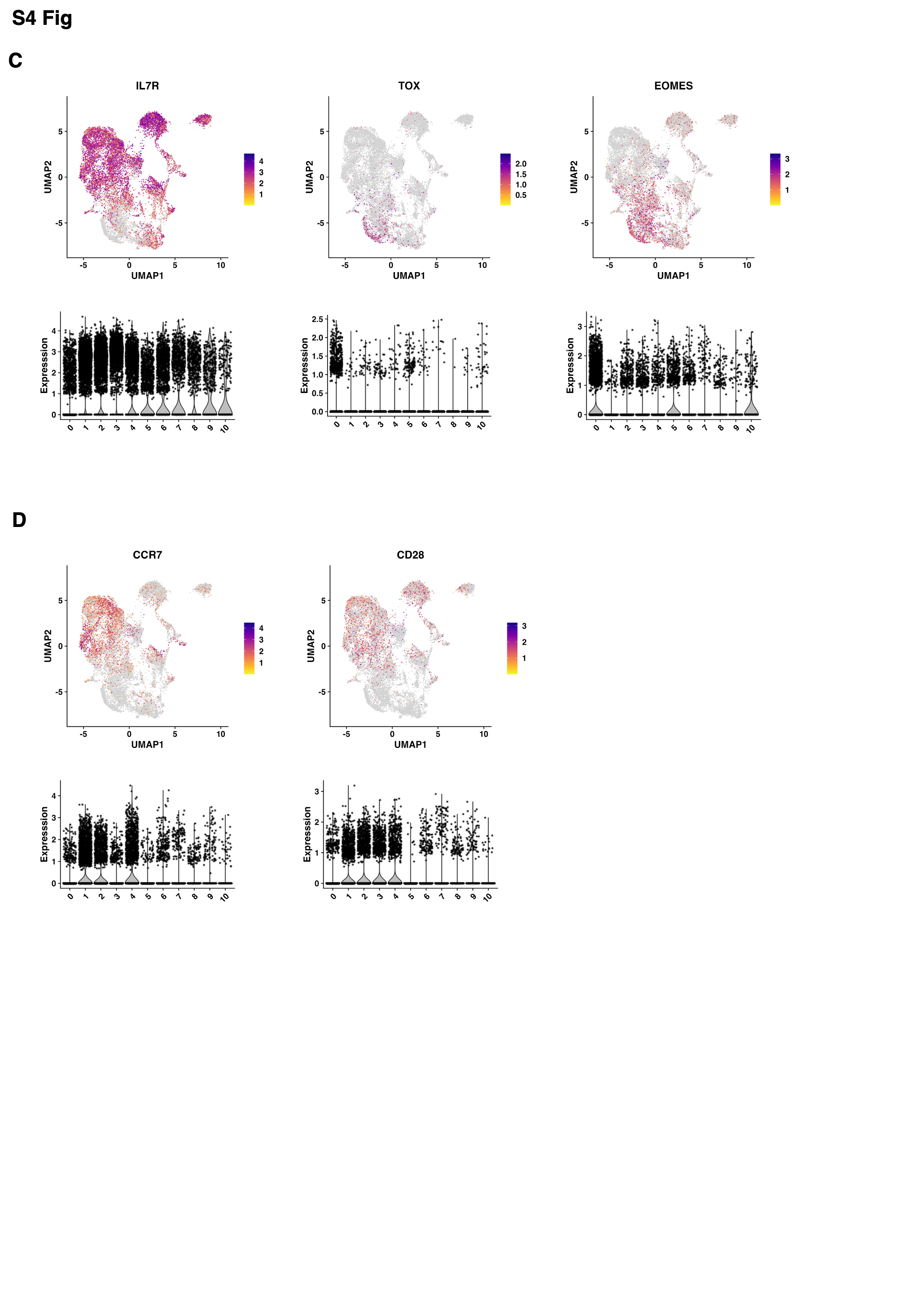

Supplement: Supplementary file 1 [file DataSheet_1.zip › Supplementary Figures 4C_D.TIFF]
