# Supplementary material for: Hepatitis B surface antigen reduction is associated with hepatitis B core-specific CD8+ T cell quality
Source: Front Immunol. 2023 Oct 18;14:1257113. doi: 10.3389/fimmu.2023.1257113 (PMC10619684; doi:10.3389/fimmu.2023.1257113)
Supplement: Supplementary file 2 [file DataSheet_2.pdf]

Supplementary Material

1 Supplementary Data

Peptide design

Overlapping peptides covering the HBs, HBcore, and HBpol regions were designed based on the consensus sequences covering genotypes A2, B2, C2, and D1 of HBV reported in Japanese patients. In total, 214 unique sequences of 10937 sequences deposited in the hepatitis virus database (http://s2as02.genes.nig.ac.jp/) were extracted as nucleic acid sequences, and the conserved sequences were converted to amino acid sequences. A 15-mer peptide with a 9-mer overlapping with the surrounding sequences was designed for each antigen. If ambiguous amino acids were located within the 15-mer sequence, each amino acid species was assigned. In total, 262, 133, and 573 peptides were synthesized from HBs, HBcore, and HBpol, respectively (Table S2). Given the small number of HBV-specific $CD8^+$ T cells in CHB patients undergoing NUC therapy, we performed *ex vivo* expansions of these cells to increase the sensitivity of cell detection. Overlapping peptides covering the consensus sequences of the HBs, HBcore, and HBpol regions of genotype A/B/C viruses were used for cell stimulation (Figure S6).

Sorting, scRNA-seq library preparation, and sequencing

Frozen PBMCs were thawed, washed with RPMI1640 medium (Sigma-Aldrich, St. Louis, MO, USA) supplemented with 10% FBS (Sigma-Aldrich), 100 U/mL of penicillin, and 100 mg/mL streptomycin (Sigma-Aldrich; hereafter referred to as R10) and treated with 1 mL benzonase (50 U/mL; Merck, Darmstadt, Germany) in R10 for 30 min at 37 °C. Cells were stained using a sorting panel (Table S3), and up to 20000 (5438 cells/CHBN001, 15987 cells/CHBN002, 16030 cells/CHBN003, 12309 cells/CHBN004, 7264 cells/CHBN005, 7240 cells/CHBN006) $CD69^+$ non-naïve activated $CD8^+$ T cells (defined by CD69/CD45RO/CD27) per donor were sorted into 20% FBS in phosphate-buffered saline (PBS). Sorted cells (purity median = 95% [range 90–96%]) were centrifuged at 1000 ×g for 5 min, washed once with PBS, centrifuged at 2000 ×*g* for 5 min, resuspended in ~17 µL of PBS, and used as input for chromium 5′-kit (10× Genomics, Pleasanton, CA, USA) beads/donor. The scRNA-seq library was produced according to the manufacturer's protocol. Libraries were combined and read using an Illumina NovaSeq 6000 platform (outsourced to AZENTA, Burlington, MA, USA).

scRNA-seq analysis and visualization

Raw sequence reads were analyzed using *cellranger* 6.1.2 (10× Genomics) with the human GRCh38 reference genome and the corresponding GTF file. Consequently, 1934, 1507, 2390, 4169, 2552, and 3021 cells were recovered from CHBN001, CHBN002, CHBN003, CHBN004, CHBN005, and CHBN006, respectively. The quantities of reads/cells were 82232, 101654, 17178, 33469, 34202, 29710 from CHBN001, CHBN002, CHBN003, CHBN004, CHBN005, and CHBN006, respectively.

Raw gene expression matrices were converted to Seurat objects. Each datum was separated, the quality was first controlled by the total number of genes detected (more than 200 genes), the frequency of mitochondrial genes (cells showing less than 10% of the mitochondrial gene frequency), and the ribosomal gene (cells showing more than 0.05% of the ribosomal gene frequency). Second, the filtered Seurat object were converted to SingleCellExperiment object and putative doublets were calculated by *scDblFinder* and removed. Lastly, gene expression levels were used to filter the remaining noisy cells (CD8A/CD8B > 0, CD3D/CD3G/CD3E >0) as in seurat objects.

After quality control, Seurat objects from each donor were merged and then integrated using the *Seurat::SelectIntegrationFeatures* (nfeatures= 3000), *PrepSCTIntegration*, *FindIntegrationAnchors* (normalization.method = "SCT") and *IntegrateData* functions. Integrated Seurat objects were normalized and clustered using the *Seurat::ScaleData*, *RunPCA*, *FindNeighbors*, *FindClusters*, and *RunUMAP* functions. Plots were generated using the *Seurat::DimPlot*, *FeaturePlot*, *VlnPlot*, and *scCustomize::FeaturePlot_scCustom* functions. Differentially expressed genes (DEGs) were identified using *Seurat::FindMarkers* by comparing Cluster-high (CL-high) and CL-low samples with the default parameters. Among the DEGs of CL-high or CL-low samples, 20 genes were selected from those with the lowest *P*-values; heatmaps were generated from the selected genes using *Seurat::DoHeatmap*. For Gene Ontology (GO) enrichment analysis, upregulated genes, which were selected as having average log2FC>0 and adjusted *P*<0.05 among the DEGs, were used as input. Enriched biological processes were extracted using *clusterProfiler::enrichGO*, and count values of the top 10 processes were visualized using bar plots with the adjusted *P*-values. For gene set enrichment analysis (GSEA), genes used for the GO enrichment analysis were selected and biological processes were extracted using *clusterProfiler::gseGO*; the top two signatures were visualized using *clusterProfiler::gseaplot*. Module scores were calculated using the *Seurat::AddModuleScore* function with selected genes shown in Table S4 for Figure 1F. Similarly, for Figure 2F, the gene list from the GSEA molecular signature datasets GOCC_CYTOLYTIC_GRANULE (systemic name: M25840) and GOBP_REGULATION_OF_LEUKOCYTE_MEDIATED_CYTOTOXICITY (systemic Name: N29012) were used.

Long-term culture and flow cytometry

Frozen PBMCs were thawed, washed with R10, and treated with 1 mL benzonase (50 U/mL; Merck, Darmstadt, Germany) in R10 for 30 min at 37 °C. Cells were cultured for 10 days in the presence of 20 U/mL recombinant IL-2 (R&D systems) to expand antigen-specific $CD8^+$ T cells, as previously described (2). Briefly, 20% of the total PBMCs were pulse-stimulated with 2 μg/mL of each peptide mixture and then cultured for 1 h. After 1 h, the remaining stimulated cells were combined with 80% of the other remaining cells and cultured in IL-2-containing R10 for 10 days. Half of the medium was replaced on days 4 and 7. After 10 days, the cells were washed with PBS and restimulated with the corresponding peptides for 6 h in R10 containing anti-CD107A antibodies. Finally, HBV-specific $CD8^+$ T cells were analyzed using flow cytometry. To prevent cytokine secretion, BFA and monensin were added to the culture after 30 min of re-stimulation.

Cell surface staining was performed after incubation as follows. First, PBMCs were washed with PBS and stained with a Live/DeadTM Fixable Aqua Dead Cell Stain Kit (L34957; Thermo Fisher) at 25 °C for 5 min. The remaining surface markers (Table S5) were added and incubated at room temperature

(RT; ~25 °C) for 15 min. After surface staining, the cells were washed twice with PBS, fixed, and permeabilized with Cytofix/Cytoperm (BD) at RT for 10 min. The cells were then washed twice with Perm/Wash (BD Biosciences) and stained with the intracellular markers listed in Table S5 at RT for 20 min. After staining, the cells were washed twice with Perm/Wash (BD Biosciences), fixed with 1% paraformaldehyde, and analyzed using flow cytometry. Flow cytometry FCS files were analyzed using FlowJo (10.8.1; BD). Antigen-specific cells were denoted by their positivity for IFN-γ, TNFα, CD107A, or CD137. The frequency of HBV-specific $CD8^+$ cells was determined by subtracting the value obtained via peptide-free stimulation (DMSO; background) from that obtained by each antigenic stimulation. After background subtraction, values ≤ 0.5% were considered negative (no response). Polyfunctionality was analyzed using Simplified Presentation of Incredibly Complex Evaluations (SPICE, version 6.1) (17).

2 Supplementary Figures and Tables

Supplementary Figure 1. CD69 and isotype control staining in naïve and memory $CD8^+$ T cells.

(A) A representative gating for naïve $CD8^+$ T cells (Tn) and memory $CD8^+$ T cells (Tm). (**B**) Representative flowcytometry plot of CD69 or isotype control staining in CD8 Tn and CD8 Tm. (**C**) Frequency of cells in CD69-positive gate in Tn and Tm.

Supplementary Figure 2. Clustering of scRNA-seq data and removal of cluster (CL) 11.

(A) Representative image of the UMAP with 12 clusters (CL0–CL11). (B) Number of cells in each cluster of each donor. Each dot indicates the data from individual donors. Crossbars indicate the median value of each cluster. (C) Frequency of cells in each cluster of each donor. Each dot indicates the data from individual donors. Colors indicate each donor. Crossbars indicate the median value of each cluster. (D) Frequency and absolute number of cells in CL11 of each donor.

Supplementary Figure 3. Frequency of each cluster in HBsAg-high or -low donors.

Dot plot indicating the frequency of each cluster in the HBsAg-high or -low donor groups. Crossbars indicate the median value. *P* values indicated were calculated by Wilcoxon rank sum test (=Mann-Whitney *U* test) implemented by *stat_compare_means* function in *ggpubr* package.

Supplementary Figure 4. Expression of selected genes in each cluster.

(A) UMAP (top) and violin (bottom) plots indicating the relative expression levels of representative cytolytic markers within each cluster. (B) Expression of representative interferon-stimulated genes enriched in the CL-low group. (C) Expression of representative genes involving terminal exhaustion and long-lived stem cell-like property of $CD8^+$ T cells. (D) Expression of representative memory markers.

Supplementary Figure 5. Genes highly expressed in each cluster.

Heatmap showing the relative expression of differentially expressed genes among the 11 distinct clusters defined using Seurat. Top 10 DEGs from each cluster were selected for the plot.

Supplementary Figure 6. Conserved amino acid sequences in the HBs, HBcore, and HBpol regions.

Alignment of HBs, HBcore, and HBpol consensus sequences from genotypes A2, B2, C2, and D1 shown as amino acid sequences. Conserved regions are shown as blue highlights.

Supplementary Figure 7. Proportion of responders and non-responders in each antigen-stimulation group.

Pie charts presenting the proportion of responders and non-responders in the indicated antigen-specific $CD8^+$ T cells. Responders were defined according to the above threshold; donors were classified as responders or non-responders based on the frequency of HBV-specific $CD8^+$ T cells above the threshold (0.5% of alive $CD8^+$ T cell subset). NR, non-responder; R, responder. The text in the slices indicates the frequency of responders and non-responders. Three gating strategies: “MFI high”, “MFI low”, and “total” used to calculate the frequencies of antigen-specific $CD8^+$ T cells are labeled on the right as “high”, “low”, and “total” respectively.

Supplementary Figure 8. Analysis of ALT or HBV.DNA levels associated with the overall pool of $CD8^+$ T cells specific to either HBs-, HB-core, or HBpol.

(A) Dotplots indicating the ALT levels in responders or non-responders, with the peptide pool used for stimulation shown on the X-axis. Cross bar indicates the median. (B) Dotplots indicating the HBV.DNA levels in responders or non-responders, with the peptide pool used for stimulation shown on the X-axis. Cross bar indicates the median. (C) Scatter plots indicating the frequency of HBV-specific cells against the indicated antigen vs. ALT level. (D) Scatter plots indicating the frequency of HBV-specific cells against the indicated antigen vs. HBV.DNA level. R, responder; NR, non-responder. The mean difference between NR and R was examined using the Mann–Whitney *U* test. Correlations were examined using Spearman’s correlation analysis. Statistical significance is indicated by *P*-values. Three gating strategies: “MFI high,”, “MFI low,” and “total” used to calculate the frequencies of antigen-specific $CD8^+$ T cells are labeled on the right or header as “high,” “low,” and “total,” respectively.

Supplementary Figure 9. Positivity of HBV-specific $CD8^+$ T cells to each marker in patients with chronic hepatitis B and the association between HBV-specific $CD8^+$ T cells and HBsAg levels.

(A) Frequencies of IFN-γ^+, TNFα^+, $CD107A^+$, or $CD137^+$ cells in $CD8^+$ T cells in each stimulation. (B) Scatter plots indicating the frequency of each marker-positive HBV-specific cell against each indicated antigen vs. the HBsAg level. (C) Scatter plots indicating the frequency of each marker-positive HBV-specific cell against each indicated antigen vs. ALT level. (D) Scatter plots indicating the frequency of each marker-positive HBV-specific cell against each indicated antigen vs. HBV.DNA level. Correlations were examined using Spearman’s correlation analysis. Statistical significance is indicated by *P*-values. Three gating strategies: “MFI high,” “MFI low,” and “total” used to calculate the frequencies of antigen-specific $CD8^+$ T cells are labeled in right or header as “high,” “low,” and “total,” respectively.

Supplementary Figure 10. Association between ALT or HBV.DNA levels and two-subpopulation of antigen-specific $CD8^+$ T cells.

(A) Correlation between the frequency of the two HBV-specific $CD8^+$ T cell subsets (as depicted in Figure 5A) and ALT levels was examined. (B) Correlation between the frequency of the two HBV-specific $CD8^+$ T cells subsets (as depicted in Figure 5A) and HBV.DNA levels was examined. The correlations were analyzed using Spearman's correlation analysis. Statistical significance is indicated by *P*-values. Three gating strategies: "MFI high," "MFI low," and "total" used to calculate the frequencies are labeled in the header as "high," "low," and "total," respectively.

Supplementary Figure 11. Side-by side comparison of antigen-specific $CD8^+$ T cells detected by *ex vivo* analysis vs post-culture analysis.

(**A**) Raw flowcytometry plots of antigen-specific $CD8^+$ T cells in each experimental condition (N=8). (**B**) Frequencies of antigen-specific $CD8^+$ T cells defined by IFN-γ and/or TNFα in each condition (N=8) (**C**) Proportion of responders in each condition (N=8).

Supplementary Table 1. List of specimens used in this study.

List of specimens used in this study with clinical information of patients enrolled in this study. The IDs, disease condition, doses, age, sex, genotypes of HBV treatment, HBV DNA, HBsAg level, HBeAg, ALT level, and availability of scRNA-seq data are summarized.

Supplementary Table 2. List of overlapping peptides used in this study.

Shown is the list of peptides used in this study with related details. Each peptide's unique ID, peptide SN in each group, HBV protein the peptide was derived from, HBV genotype, and sequence are summarized. As the table contains a long list, it is provided as a separate spreadsheet file.

Supplementary Table 3. FACS panel used for cell sorting.

Flow cytometry panel used to sort $CD69^+$ non-naïve activated $CD8^+$ T cells. The column name "Staining" indicates the staining protocol described in the Materials and Methods section.

Supplementary Table 4. List of genes used for gene signature analysis.

List of genes and their functional categories used for the gene signature analysis. Owing to its length, this list is provided as a separate spreadsheet file.

Supplementary Table 5. FACS panel for HBV-specific $CD8^+$ T cells.

Flow cytometry panel used to detect HBV-specific $CD8^+$ T cells after long-term cell culture. The column name "Staining" indicates the staining protocol described in the Materials and Methods section.

Supplementary Table 6. R/Bioconductor packages used in this study.
